# miRNA Signature of Mouse Helper T Cell Hyper-Proliferation

**DOI:** 10.1371/journal.pone.0066709

**Published:** 2013-06-25

**Authors:** Connie L. Sommers, Alexandre K. Rouquette-Jazdanian, Ana I. Robles, Robert L. Kortum, Robert K. Merrill, Wenmei Li, Nandan Nath, Elizabeth Wohlfert, Katherine M. Sixt, Yasmine Belkaid, Lawrence E. Samelson

**Affiliations:** 1 Laboratory of Cellular and Molecular Biology, Center for Cancer Research, National Cancer Institute, National Institutes of Health, Bethesda, Maryland, United States of America; 2 Laboratory of Human Carcinogenesis, Center for Cancer Research, National Cancer Institute, National Institutes of Health, Bethesda, Maryland, United States of America; 3 Mucosal Immunology Section, Laboratory of Parasitic Diseases, National Institute of Allergy and Infectious Disease, National Institutes of Health, Bethesda, Maryland, United States of America; Maisonneuve-Rosemont Hospital, Canada

## Abstract

Helper T cells from a mutant mouse model, LAT Y136F, hyper-proliferate and cause a severe lymphoproliferative disease that kills the mice by six months of age. LAT Y136F mice carry a tyrosine to phenylalanine mutation in the Linker for Activation of T cells (LAT) gene. This mutation leads to a number of changes in T cells that result in altered cytokine production including increased IL-4 production, increased proliferation, and decreased apoptosis. Hyper-proliferation of the mutant T cells contributes to lymphadenopathy, splenomegaly, and multi-organ T cell infiltration. miRNAs are short non-coding RNAs that regulate expression of cohorts of genes. This study investigates which miRNAs are expressed in LAT Y136F T cells and compares these to miRNAs expressed in wild type T cells that are undergoing proliferation in two other settings. The first setting is homeostatic proliferation, which was modeled by adoptive transfer of wild type T cells into T cell-deficient mice. The second setting is proliferation in response to infection, which was modeled by infection of wild type mice with the nematode *H. polygyrus*. By comparing miRNA expression in these three proliferative states (LAT Y136F hyper-proliferation, homeostatic proliferation and proliferation in response to *H. polygyrus* infection) to expression in wild type naïve CD4^+^ T cells, we found miRNAs that were highly regulated in all three proliferative states (miR-21 and miR-146a) and some that were more specific to individual settings of proliferation such as those more specific for LAT Y136F lymphoproliferative disease (miR-669f, miR-155 and miR-466a/b). Future experiments that modulate levels of the miRNAs identified in this study may reveal the roles of these miRNAs in T cell proliferation and/or lymphoproliferative disease.

## Introduction

During an immune response to an infectious agent, a few, rare T cells specific for a particular antigen are activated and proliferate. T cell proliferation is first initiated by binding of foreign antigen presented by a major histocompatibility complex molecule to the T cell antigen receptor (TCR). This binding event, along with engagement of other cell surface receptors, leads to the activation of various transcription factors through the action of signaling mediators. In turn, this leads to the transcription of genes involved in proliferation including cytokine genes. T cell proliferation continues over days. When antigen-specific T cells are no longer needed after the infection is cleared, most die while a few become long-lived, quiescent memory T cells. With the combined action of suppressive mechanisms, T cells return to a “normal” number and distribution [1], [2], [3].

We have described a mutant mouse model wherein T cells hyper-proliferate in an ongoing manner in the absence of infection or other usual proliferation triggers. The proliferating T cells are a polyclonal population of CD4^+^ T helper cells. They have a Th2 phenotype, producing a particular subset of cytokines including IL-4. T cell numbers increase over time in these mutant mice and this abnormal T cell proliferation causes enlargement of peripheral lymphoid organs. T cell numbers can reach ten-fold higher than normal and the mutant T cells have defective rates of TCR-induced apoptosis. T cells infiltrate into multiple other organs, including lungs, leading to death of the mice by six months of age. The mutation that gives rise to this phenotype is a tyrosine to phenylalanine substitution in the Linker for Activation of T cells gene (LAT Y136F) [4], [5]. This mutation prevents binding of PLC-γ1 to LAT, which is needed for downstream TCR-induced calcium flux and normal T cell activation [4], [5] but paradoxically activates signaling pathways upstream of the MAP kinase Erk [6], [7], [8].

Hyper-proliferative LAT Y136F mutant T cells share characteristics with two different proliferative states induced in wild type CD4^+^ T cells. In both of these states wild type T cells proliferate in response to a trigger, and then T cell numbers return to normal. The first state is homeostatic proliferation, the process by which T cells proliferate in a T cell-depleted environment such that normal T cell numbers can be restored and maintained. Homeostatic proliferation may operate in many instances in which T cells numbers are depressed, for example, during aging as thymic involution occurs or after transfer of T cells to a T cell-deficient environment [9], [10]. In this study, we modeled homeostatic proliferation by adoptively transferring wild type CD4^+^ T cells to recipients that lack αβ T cells because of a genetic deficiency in the LAT gene. After homeostatic expansion, constant T cell numbers are maintained by a balance of proliferation and apoptosis. Homeostatic proliferation is important clinically as this process restores T cell numbers in patients whose hematopoietic cells have been ablated by radiation or chemotherapy in preparation for transfer of normal or genetically engineered hematopoietic cells [11]. Proliferation of LAT Y136F T cells is similar to homeostatic proliferation during adoptive transfer to immunodeficient animals in that it is IL-7-dependent and initially occurs in a reduced T cell compartment [12]. Also, hyper-proliferation of LAT Y136F T cells is thought to be dependent on development in a T cell-deficient environment because LAT Y136F mutant T cells do not cause lymphoproliferative disease in the presence of T cells expressing wild type LAT (e.g. in heterozygous mice [4], [5], [13]) and do not cause lymphoproliferative disease when adoptively transferred with T cells expressing wild type LAT [12]. The second proliferative state is proliferation in response to helminth infection. Infection with *H. polygyrus* results in activation and proliferation of T cells with a Th2 phenotype [14], which are phenotypically similar to the continuously proliferating Th2-type cells found in LAT Y136F mice.

In an effort to study how T cell hyper-proliferation occurs in LAT Y136F mice compared to more well-regulated proliferative states, we sought to identify what genes might regulate a hypothetical LAT Y136F T cell “proliferative program”. MicroRNAs (miRNAs) are small (approximately 22 nucleotide) RNAs that modulate the expression of genes for which they have homology, most importantly in the central “seed” region of the miRNA [15]. A single miRNA can influence the expression of many (possibly hundreds) of genes. Therefore, if the regulated genes are important for a particular phenotype, changes in miRNA levels could direct cells toward a certain differentiation or functional pathway. A few of many examples include miRNAs involved in inflammation (miR-146a) [16], autoimmune disorders (miR-21, miR-155) [17], and cancer (miR-10b, let-7) [18].

In this study, we have examined miRNA levels in T cells proliferating during a normal immune response to helminth infection, during homeostatic proliferation, and in LAT Y136F lymphoproliferative disease in an effort to identify miRNAs that direct hyper-proliferation in LAT Y136F T cells and that are involved in helper T cell proliferation in general.

## Materials and Methods

### Mice and Ethics Statement

Mice were maintained under pathogen-free conditions at American Association for the Accreditation of Laboratory Animal Care-accredited facilities at the NCI and NIAID (*H. polygyrus* infection). Mice were housed in accordance with the recommendations in the Guide for the Care and Use of Laboratory Animals of the National Institutes of Health under animal study proposals approved by the NCI-Bethesda Animal Care and Use Committee (ASP#LCMB-013) and the NIAID Animal Care and Use Committee (ASP#LPD-7E). C57BL/6 mice were obtained from the NCI/DCTD/DTP/BTB Animal Production Program (Frederick, MD). LAT Y136F knock-in and LAT knockout mice (αβ T cell-deficient) were generated in our laboratory [4], [19] and were backcrossed more than 10 generations onto C57BL/6. The LAT Y136F mouse used for Nanostring analysis of miRNA expression was 11 weeks old.

### Flow Cytometry and Cell Sorting

Standard flow cytometry was performed using a FACSCalibur (BD Biosciences) and FlowJo software (Tree Star Inc.). Cell sorting was performed using a BD FACSAria II (BD Biosciences) by the NCI Flow Cytometry Core. The following antibodies were purchased from BD Biosciences: CD4 (GK1.5), CD8 (53–6.7), CD25 (PC61), CD44 (IM7) and CD62L (MEL-14).

### Homeostatic Proliferation

For adoptive transfer/homeostatic proliferation experiments, LAT knockout mice were lightly anesthetized by isofluorane inhalation and 1×10^6^ sorted T cells from C57BL/6 mice were adoptively transferred by retro-orbital injection. Mice were monitored during and after the procedure for signs of distress or illness. 17 days after adoptive transfer, lymph nodes and spleen were harvested and CD4^+^ T cells were purified by sorting. 17 days was chosen for analysis because it was the earliest time post-transfer that allowed for RNA isolation in adequate amounts for analysis while cells were still undergoing homeostatic proliferation as measured by BrdU incorporation.

### *H. polygyrus* Infection Protocol

Mice were perorally infected with 200 *Heligmosomoides polygyrus* infective larvae (L3 stage) as previously described [20]. 23 days post infection, spleens and mesenteric lymph nodes were harvested and CD4^+^CD44^hi^ T cells were purified by sorting.

### RNA Isolation and Nanostring Analysis of miRNA Expression

RNA was isolated from sorted T cells using the mirVana miRNA isolation kit (Invitrogen). miRNA expression levels were determined by using the nCounter® mouse miRNA expression assay kit (Nanostring Technologies) and the nCounter® analysis system (Nanostring Technologies, courtesy of the NCI DNA Sequencing Core). Data were analyzed using nSolver Analysis Software (Nanostring Technologies) and Partek Genomics Suite 6.6 software (Partek Inc.). MicroRNA probes were deleted if signal intensity <15 in >50% samples, leaving 92 probes. In addition, “dead” miRNAs according to miRBase 19 were deleted, leaving 86 miRNAs. Data were log2-transformed. Exploratory analyses were conducted by Unsupervised Hierarchical Clustering, and miRNA expression differences were evaluated on log2-transformed intensity values using intensity ratios. For pathway analysis, targets of the 86 miRNAs expressed above the minimum intensity threshold were interrogated by PubMed search. The targets were then classified into pathways known to be important for T cell signaling and development.

### Reverse Transcription-Quantitative PCR

Reverse transcription: All reagents for reverse transcription and qPCR reactions were obtained from Life Technologies. 10 ng total RNA was reverse transcribed using TaqMan® MicroRNA Reverse Transcription Kit and gene-specific primers, hsa-miR-21, hsa-miR-146a, hsa-miR-148a, hsa-miR-181a, and snoRNA202. After reverse transcription, qPCR was performed using TaqMan® Universal PCR Master Mix II (No UNG), cDNA, and TaqMan® hydrolysis probes, hsa-miR-21, hsa-miR-146a, hsa-miR-148a, hsa-miR-181a, and snoRNA202. qPCR amplification was performed on ABI 7900HT machine in 10 µl reactions. qPCR Analysis: Samples were amplified with four experimental replicates, each from pools of four to eight mice or from individual mice for LAT Y136F mice (biological replicates). No template controls were reliably negative. All samples were compared to the reference gene, sno202, which did not show significant variability across samples. The mean reference gene Cq for all biological and experimental replicates was subtracted from each target miRNA Cq to achieve the dCq. The mean and standard deviation of these was used to achieve ddCq values and fold change between LAT Y136F and wild type (C57BL/6) naïve cells CD4^+^ T cells, between wild type memory and wild type naïve CD4^+^ T cells, and between LAT Y136F and wild type memory CD4^+^ T cells was calculated from 2?-(ddCq). Standard deviation was propagated, and standard error was calculated finally to achieve upper and lower error bars. Statistical Analysis: One-way ANOVA (Analysis Of Variance) was performed to determine biological variation for biological replicates for each miRNA within a given condition. C57BL/6 naïve CD4^+^ T cells, C57BL/6 memory CD4^+^ T cells, and LAT Y136F CD4^+^ T cells showed non-significant variation between mice or pools of mice for most miRNAs. However, miR-148a showed small variation between mice within all conditions. *P*-values and *F*-values were obtained, and *Fcrit* was determined for α = 0.05. Two-way ANOVA with replication was performed to determine significance in fold-change between LAT Y136F and C57BL/6 naïve CD4^+^ T cells, between C57BL/6 memory and C57BL/6 naïve CD4^+^ T cells, and between LAT Y136F and C57BL/6 memory CD4^+^ T cells. *P*-values and *F*-values were obtained, and *Fcrit* was determined for α = 0.05.

## Results and Discussion

In this study, we investigated miRNAs regulated in CD4^+^ helper T cell proliferative states as well as identified miRNAs that potentially control hyper-proliferation in mutant LAT Y136F T cells. As a basis for comparison, we first purified and analyzed miRNAs from naïve, unactivated CD4^+^ helper T cells from wild type (C57BL/6) mice. Naïve CD4^+^ T cells display the cell surface marker pattern CD4^+^CD25^-^CD44^lo^CD62L^hi^. Naïve CD4^+^ T cells were purified by sorting from C57BL/6 mice and RNA was made from the sorted population. Expression levels of individual miRNAs as measured by Nanostring analysis in naïve C57BL/6 CD4^+^ T cells are shown in Figure S1 and Table S7. Out of 565 endogenous miRNAs measured, we focused on the highest expressing 15% (Nanostring counts >15). Although we present levels of miRNA expression determined by Nanostring nCounter technology here, expression levels of some samples were also confirmed by Exiqon microarray analysis (data not shown) and by Taqman qRT-PCR analysis (discussed below). In the literature, miRNA profiling studies of mouse CD8 naïve T cells are more abundant than those of CD4 naïve T cells. However, comparison of the seven most highly expressed miRNAs in CD8^+^ T cells with the seven most highly expressed miRNAs in naïve CD4^+^ T cells from this study reveal four in common: miR-150, miR-142-3p, miR-16 and miR-15b [21].

LAT Y136F mice contain hyper-proliferative T cells and expansion of these T cells leads to lymphadenopathy, splenomegaly, and infiltration into lung, liver, kidney and multiple other organs. To investigate what miRNAs could be involved the hyper-proliferative state of these T cells, we compared their miRNA expression to miRNA expression in naïve, wild type C57BL/6 CD4^+^ T cells. Table 1 summarizes fold changes of miRNA expression among 86 of the miRNAs most highly expressed in naïve C57BL/6 CD4^+^ T cells (approximately 15% of all miRNAs screened; see Table S1 for fold change values). miRNAs are listed from top to bottom in order of decreasing expression in wild type naïve CD4^+^ T cells. Arrows indicate fold increases (up arrow) or decreases (down arrow). One arrow indicates a greater than 2-fold difference and two arrows a greater than 5-fold difference in expression. We noted many striking differences in miRNA expression levels between LAT Y136F CD4^+^ T cells and C57BL/6 naïve CD4^+^ T cells. Four miRNAs (miR-21, miR-181a, miR-146a and miR-148a) have a greater than 10-fold difference in expression between LAT Y136F and naïve C57BL/6 CD4^+^ T cells and five additional miRNAs have greater than 5-fold differences (Table 1; miR-669f, miR-155, miR-466a/b, miR-125a and miR-96). Since even 2-fold differences in miRNA levels can lead to phenotypic changes, there are many miRNAs from this comparison that merit future follow-up analysis.

**Table 1:** Fold Changes in miRNA expression relative to B6 naïve CD4^+^ T cells*.

miRNA	LAT Y136F	B6 HP	B6 H poly	B6 memory
mmu-miR-150		↓		↓
mmu-miR-15b	↓			
mmu-miR-30b		↓		
mmu-let-7f		↓		
mmu-miR-21	↑↑	↑↑	↑↑	↑↑
mmu-let-7c	↓	↓		
mmu-miR-181a	↓↓	↓↓		
mmu-miR-15a	↑	↑	↑	
mmu-let-7b	↓	↓		↓
mmu-miR-342-3p		↓		
mmu-miR-26b		↓		
mmu-miR-669f	↓↓	↓	↓	
mmu-miR-151-3p		↓	↓	↓
mmu-miR-29c	↑			
mmu-miR-151-5p		↓		↓
mmu-miR-155	↑↑	↑	↑	↑↑
mmu-miR-467f	↓	↓		
mmu-miR-378	↓	↓	↓	
mmu-miR-466a/b-3p	↓↓	↓	↓	
mmu-miR-146a	↑↑	↑↑	↑↑	↑↑
mmu-miR-145			↓	
mmu-miR-10a	↓	↓	↓	
mmu-miR-27a	↑	↑	↑	↑
mmu-miR-361		↓		
mmu-miR-22	↑	↑	↑	↑
mmu-miR-423-5p		↓		
mmu-miR-547	↓			
mmu-miR-338-5p			↓	
mmu-let-7i	↑			
mmu-miR-883b-3p				↓
mmu-miR-1949	↓	↓		
mmu-miR-340-5p	↓			
mmu-miR-544			↓	
mmu-miR-148a	↑↑	↑↑	↑	↑
mmu-miR-139-5p	↓			
mmu-miR-132	↑			
mmu-miR-23a	↑	↑	↑	↑
mmu-miR-539		↑		
mmu-miR-93		↑		
mmu-miR-125a-5p	↑↑		↑	↑
mmu-miR-130b	↑			
mmu-let-7e	↑			
mmu-miR-350				↑
mmu-miR-24	↑	↑	↑	↑
mmu-miR-107	↑	↑	↑	↑
mmu-miR-1902	↑	↑	↑	↑
mmu-miR-96	↑↑	↑↑	↑	

* miRNAs with Nanostring counts that passed the minimum intensity filter and have fold changes relative to C57BL/6 naïve CD4^+^ T cells >2-fold up (↑), >5-fold up (↑↑), >2-fold down (↓) or >5-fold down (↓↓). miRNAs are ordered by rows according to expression in C57BL/6 naïve CD4^+^ T cells beginning with highest expression on the top. Exact values of fold changes can be found in Table S1. LAT Y136F indicates LAT Y136F CD4^+^ T cells, B6 HP indicates C57BL/6 CD4^+^ T cells undergoing homeostatic proliferation, B6 H poly indicates C57BL/6 CD4^+^ T cells from *H. polygyrus*-infected mice and B6 memory indicates C57BL/6 CD4^+^ T cells that are also CD44^hi^CD62L^lo^. B6 naïve CD4^+^ T cells are C57BL/6 CD4^+^ T cells that are also CD44^lo^CD62L^hi^.

Homeostatic proliferation ensues after adoptive transfer of wild type T cells to T cell-deficient recipients. Although the transferred T cells rapidly proliferate, they do not continue to expand uncontrollably, but reach a “normal” T cell number that is maintained over time by a balance of proliferation and cell death. This is in contrast to LAT Y136F hyperproliferating T cells, which continue to expand beyond normal numbers. To investigate miRNA expression in T cells undergoing homeostatic proliferation, we adoptively transferred sorted CD4^+^ lymphocytes from C57BL/6 mice to LAT-deficient mice, which do not have αβ T cells. In this situation, the adoptively transferred T cells rapidly proliferate. 17 days after adoptive transfer, the expanded CD4^+^ T cells of donor origin were purified by sorting from the recipient mice. RNA was extracted and analyzed for miRNA expression. Three miRNAs showed more than 10-fold differences of expression in CD4^+^ T cells from mice undergoing homeostatic proliferation compared to naïve C57BL/6 CD4^+^ T cells (miR-21, miR-181a and miR-146a, Table S1) and two additional miRNAs showed greater than 5-fold differences (miR148a and miR-96, Table 1).

Because LAT Y136F T cells are Th2-like, IL-4 producing cells, for comparison we chose an infection model to study that also elicits a robust Th2 response, infection with *H. polygyrus* nematodes. Mice were infected and 23 days post-infection, CD4^+^CD44^hi^ T cells were analyzed for miRNA expression. In comparing to T cells from naïve wild type mice, two miRNAs were overexpressed more that 10-fold (miR-21 and miR-146a, Table S1).

**Figure 1:**
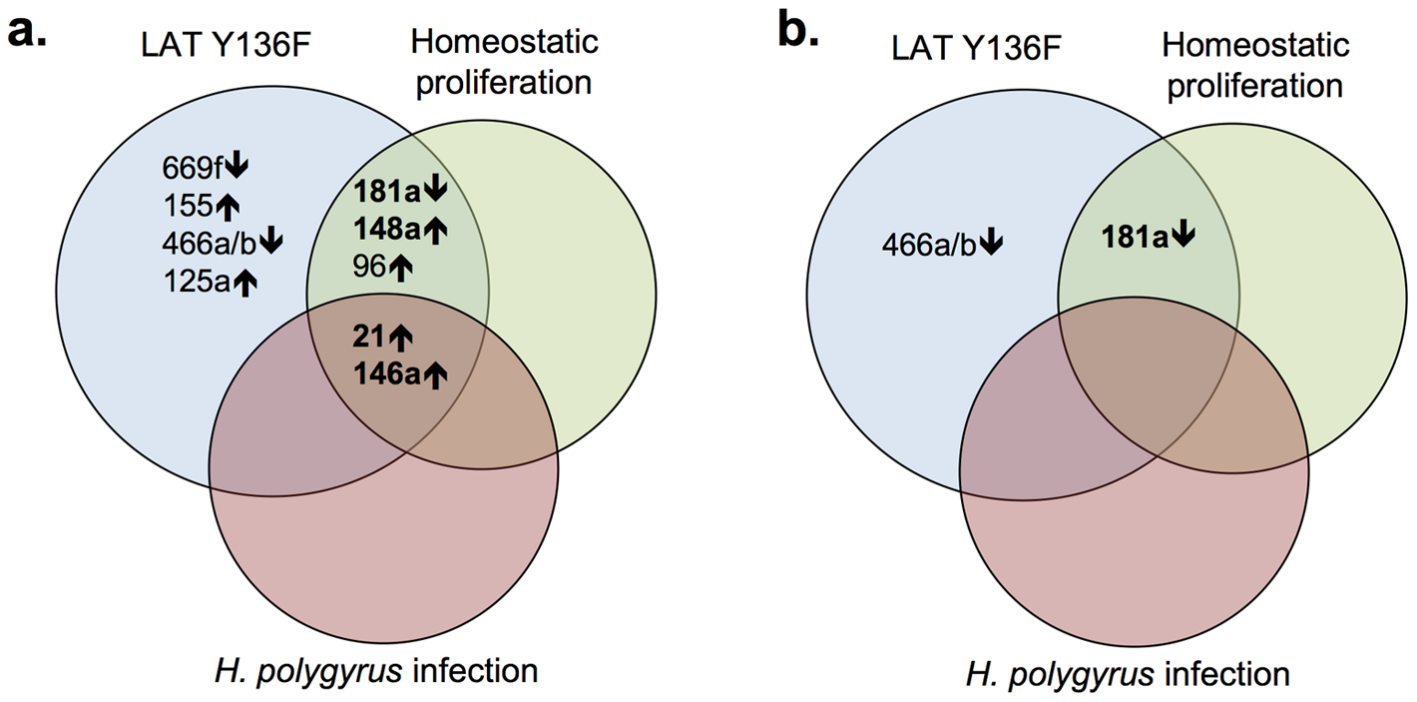
miRNAs that are most highly regulated in proliferating CD4^+^ T cells. a. miRNAs that are increased (↑) or decreased (↓) in expression more than 5-fold relative to C57BL/6 naïve CD4^+^ T cells. miRNAs that are increased or decreased in LAT Y136F CD4^+^ T cells more than 10-fold are shown in bold. b. miRNAs that are increased (↑) or decreased (↓) in expression more than 5-fold relative to C57BL/6 memory CD4^+^ T cells.

The Venn diagram in Figure 1a depicts miRNAs expressed in proliferating CD4^+^ T cells at levels greater than 5-fold different from those in naïve wild type CD4^+^ T cells (greater than 10-fold differences are shown in bold). The two miRNAs differentially over-expressed in all three proliferative states were miR-21 and miR-146a. Three miRNAs had altered expression in LAT Y136F CD4^+^ T cells and in CD4^+^ T cells undergoing homeostatic proliferation (miR-181a, miR148a and miR-96). Of the three different types of proliferating CD4^+^ T cells, LAT Y136F CD4^+^ T cells had altered expression of miRNAs not seen in the other two types of T cells. These miRNAs differentially regulated in LAT Y136F CD4^+^ T cells were miR-669f, miR-155, miR466a/b and miR-125a.

In each of the three proliferative states described above, the proliferating T cells had an “activated” phenotype, i.e. they expressed the same markers as memory T cells, which have previously been activated by exposure to antigen, have proliferated and have entered into a quiescent, memory state (and are CD44^hi^CD62L^lo^). Therefore, in an effort to identify miRNAs that correlated with an activated/memory state, we compared miRNA expression in wild type C57BL/6 memory CD4^+^ T cells and wild type C57BL/6 naïve CD4^+^ T cells. As shown in the final column of Table 1, three miRNAs showed greater than 10-fold differences (miR-21, miR-155 and miR-146a). These miRNAs are potentially involved in activation, proliferation or memory T cell development.

We next directly compared miRNA expression from T cells from the three proliferative states described above to miRNA expression in C57BL/6 memory CD4^+^ T cells, perhaps a more relevant comparison than to naïve T cells (Table 2 and Table S2). In contrast to comparisons to C57BL/6 naïve CD4^+^ T cells (also see Figure 1b), fewer miRNAs were differentially regulated, particularly by 5-fold or more. In hyper-proliferating LAT Y136F T cells, one miRNA (miR-181a) was underexpressed more than 10-fold compared to C57BL/6 memory CD4^+^ T cells and another was underexpressed more than 5-fold (miR-466a/b). In the case of homeostatic proliferation, miR-181a was also underexpressed more than 10-fold relative to C57BL/6 memory CD4^+^ T cells. Of the three proliferative states, T cells from mice previously infected with *H. polygyrus* showed the most similar pattern of miRNA expression compared to wild type C57BL/6 memory CD4^+^ T cells. miR-181a was dramatically downregulated in LAT Y136F T cells and in T cells undergoing homeostatic proliferation in comparison to wild type memory T cells. Whether underexpression of miR-181a is a marker or cause of active proliferation in these settings remains to be determined. That all three proliferative states showed fewer miRNAs differentially expressed relative to wild type memory T cells than to wild type naïve T cells reflects the fact that these proliferating cells are phenotypically more like memory than naïve T cells.

**Table 2:** Fold Changes in miRNA expression relative to B6 memory CD4^+^ T cells*.

miRNA	LATY136F	B6 HP	B6 H poly
mmu-miR-150	↑		
mmu-miR-15b	↓	↓	
mmu-let-7c		↓	
mmu-miR-181a	↓↓	↓↓	
mmu-miR-26b		↓	
mmu-miR-669f	↓		
mmu-miR-151-3p	↑		
mmu-miR-297c		↑	
mmu-miR-151-5p	↑		
mmu-miR-155		↓	↓
mmu-miR-467f	↓		
mmu-miR-466a/b-3p	↓↓		
mmu-miR-361	↑	↓	
mmu-miR-423-5p		↓	
mmu-miR-883b-3p		↑	
mmu-miR-374	↑	↑	
mmu-miR-1949	↓		
mmu-miR-345-3p		↑	
mmu-miR-101b	↑		
mmu-miR-148a	↑	↑	
mmu-miR-139-5p	↓		
mmu-miR-489		↑	
mmu-miR-132		↓	
mmu-miR-93		↑	
mmu-miR-125a-5p	↑	↓	
mmu-miR-1902			↑
mmu-miR-96	↑	↑	

* miRNAs with Nanostring counts that passed the minimum intensity filter and have fold changes relative to C57BL/6 memory CD4^+^ T cells >2-fold up (↑), >5-fold up (↑↑), >2-fold down (↓) or >5-fold down (↓↓). miRNAs are ordered by rows according to expression in C57BL/6 naïve CD4^+^ T cells beginning with highest expression on the top. Exact values of fold changes can be found in Table S2. LAT Y136F indicates LAT Y136F CD4^+^ T cells, B6 HP indicates C57BL/6 CD4^+^ T cells undergoing homeostatic proliferation and B6 H poly indicates C57BL/6 CD4^+^ T cells from *H. polygyrus*-infected mice. B6 memory CD4^+^ T cells indicates C57BL/6 CD4^+^ T cells that are also CD44^hi^CD62L^lo^.

**Figure 2:**
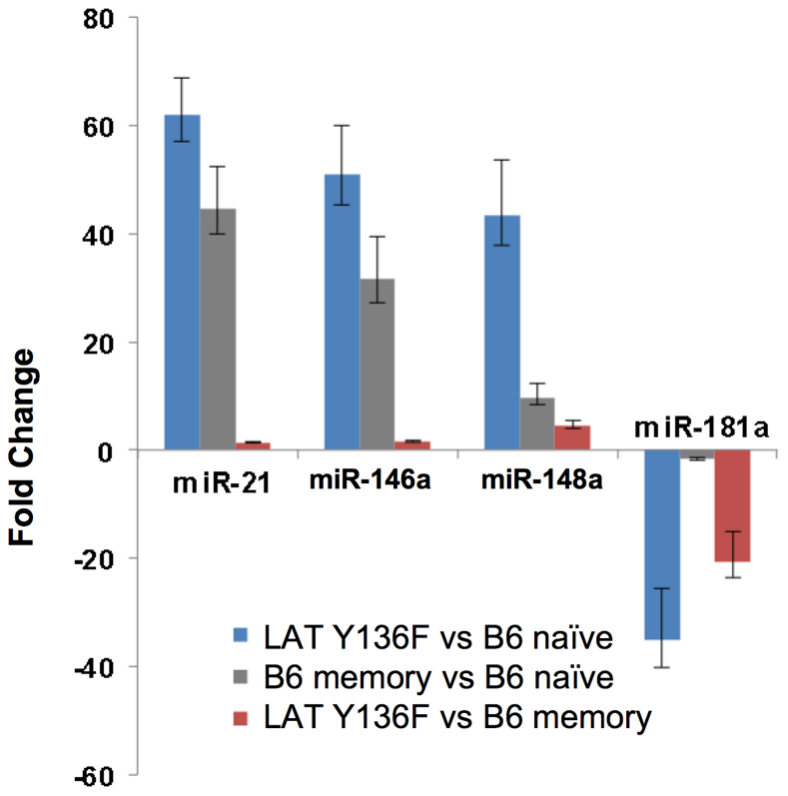
miRNA expression levels among replicate samples. RNA from sorted CD4^+^ T cells from replicate samples (RNA from sorted CD4^+^ T cells from n = 3 groups of 5 to 8 pooled C57BL/6 mice or from n = 4 individual LAT Y136F mice) was reverse transcribed and subjected in quadruplicate to qPCR analysis as described in Materials and Methods. Bars represent fold changes of LAT Y136F vs C57BL/6 naïve (blue bars), C57BL/6 memory vs C57BL/6 naïve (gray bars) and LAT Y136F vs C57BL/6 naïve (red bars) with upper and lower error bars indicating error between individual mice or groups of mice. Relevant *P-*values can be found in Table S3.

Since the analysis of miRNA levels described above relied heavily on Nanostring technology and comparison to single wild type naïve or memory CD4^+^ T cell samples, we also analyzed miRNA levels from multiple samples using reverse transcription/quantitative PCR/Taqman analysis. RNAs from three groups of wild type mice (each containing sorted naïve or memory CD4^+^ T cells from pools of five to eight C57BL/6 mice per group) and four individual LAT Y136F mice were analyzed for levels of miR-21, miR-146a, miR148a and miR-181a (miRNAs with more than ten-fold differences in expression between LAT Y136F and C57BL/6 naïve CD4^+^ T cells). The analysis was performed in quadruplicate and statistical analyses were performed for comparisons between groups (wild type naïve, wild type memory and LAT Y136F CD4^+^ T cells) or for comparing biological replicates of individual mice or pools of mice within groups (See Figure 2 for results and Table S3 for *P-* values). Two-way ANOVA with replication showed that the differences between levels of all four miRNAs in LAT Y136F CD4^+^ T cells compared to naïve wild type CD4^+^ T cells were very significant and were similar to those observed using Nanostring analysis. Wild type memory CD4^+^ T cells also differed significantly from wild type naive CD4^+^ T cells for the four miRNAs examined. For biological replicates within groups, the samples did not differ significantly by one-way ANOVA among different mice or pools of mice, with the exception of miR148a. This analysis confirmed the trends observed previously in the Nanostring analysis (i.e. overexpression of miR-21, miR-146a and miR-148a and underexpression of miR-181a in LAT Y136F CD4^+^ T cells compared to wild type CD4^+^ T cells) using a different method and multiple replicates.

**Figure 3:**
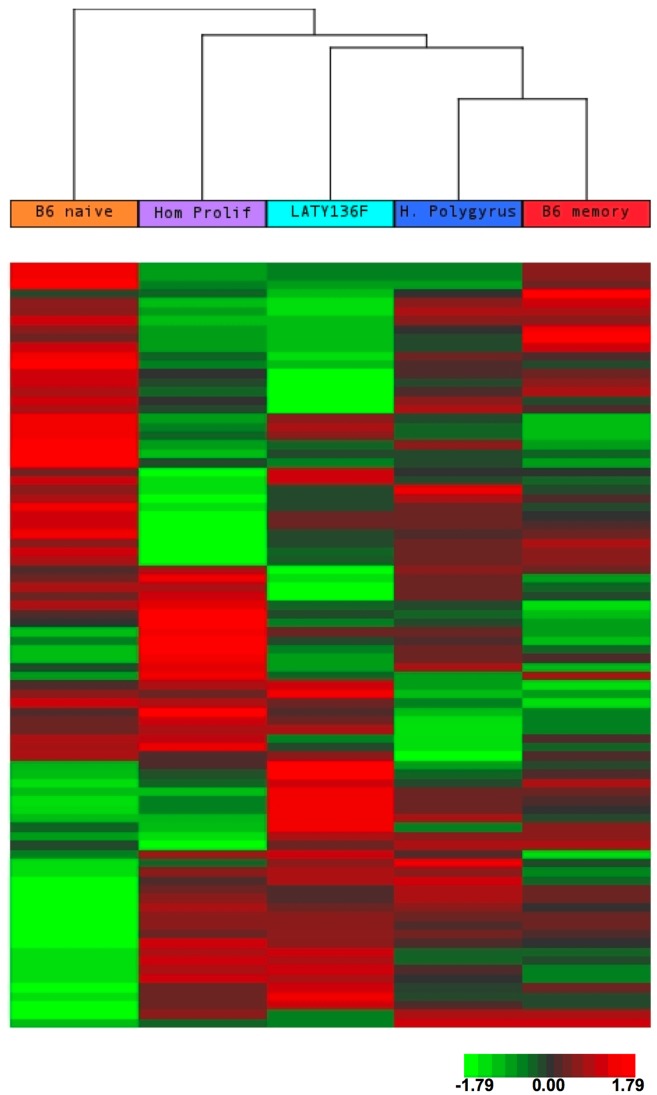
Unsupervised cluster analysis of miRNA expression among proliferating CD4^+^ T cells and CD4^+^ T cells from C57BL/6 mice. miRNA expression in T cells undergoing proliferation during helminth infection, lymphoproliferative disease, and homeostatic proliferation are compared to miRNA expression in naïve and memory T cells from wild type, C57BL/6 mice. 86 miRNAs (in rows, listed in Table S5) that passed the minimum intensity filter are clustered. Probes were median-centered and clustering was done using Euclidean distance and average linkage. Red indicates higher expression, green indicates lower expression, with respect to median.

Unsupervised clustering of all microRNAs allows visualization of overall similarities across samples (Figure 3 and Figure S2). In summary, all three proliferative states showed differential expression of miRNAs relative to wild type naïve and memory T cells, but showed more similarities to wild type memory T cells by cluster analysis (Figure 3). In other words, the wild type naïve T cells were the most “different” among the five samples. In analyzing the miRNA expression pattern of the three proliferative states (Figure S2), we found that the pattern of miRNA expression was similar in activated T cells from *H. polygyrus*-infected mice and in T cells undergoing homeostatic proliferation. The miRNA expression pattern in LAT Y136F CD4^+^ T cells had about an equivalent degree of similarity to activated T cells from *H. polygyrus*-infected mice and to T cells undergoing homeostatic proliferation. Similarities between hyper-proliferative LAT Y136F CD4^+^ T cells and T cells undergoing homeostatic proliferation is expected because both settings involve IL-7 dependence and dependence on an immunodeficient environment [12]. Roughly equivalent levels of similarity of miRNA expression in LAT Y136F CD4^+^ T cells to expression in CD4^+^ memory T cells from *H. polygyrus*-infected mice was somewhat unexpected, but may represent commonalities that result from previous T cell activation.

Lastly, we wanted to examine individual miRNA expression comparing the three different proliferative states to each other as well as to naïve and memory C57BL/6 CD4^+^ T cells (Table 3 and Tables S4 and S5). Comparison of miRNA expression from hyper-proliferative LAT Y136F T cells to T cells undergoing homeostatic proliferation might reveal miRNAs involved in maintenance of normal T cell numbers because LAT Y136 CD4^+^ T cells proliferate in a manner similar to homeostatic proliferation but continue to proliferate even after normal T cell numbers are reached. Three miRNAs were expressed at levels more than 5-fold different between LAT Y136F CD4^+^ T cells and wild type CD4^+^ T cells undergoing homeostatic proliferation (miR-361, miR-139 and miR-125). These three miRNAs may be involved in T cell expansion beyond normal cell numbers. Comparison of hyper-proliferative LAT Y136F T cells to T cells from mice previously infected with *H. polygyrus* showed one miRNA with a greater than 5- or 10-fold difference, miR-181a. In addition, comparison of miRNAs from CD4^+^ T cells undergoing homeostatic proliferation to those from *H. polygyrus*-infected mice showed one miRNA with a greater than 5- or 10-fold difference, miR-181a. Because miR-181a was dramatically underexpressed in LAT Y136F CD4^+^ T cells and in T cells from mice undergoing homeostatic proliferation compared to CD4^+^ T cells from *H. polygyrus*-infected mice, it may be involved in T cell expansion in T cell-depleted environments. Future studies could address if these miRNAs have such functions.

**Table 3:** Comparison of miRNA expression in three proliferation model systems*.

miRNA	LAT Y136F vs. HP	LAT Y136F vs. H poly	HP vs. H poly
mmu-miR-150	↑		
mmu-miR-181a		↓↓	↓↓
mmu-miR-669f	↓	↓	
mmu-miR-29c	↑		
mmu-miR-155	↑	↑	
mmu-miR-467f		↓	
mmu-miR-466a/b-3p	↓	↓	
mmu-miR-361	↑↑		↓
mmu-miR-547	↓		
mmu-miR-1949		↓	
mmu-miR-345-3p	↓		↑
mmu-miR-101b		↑	
mmu-miR-340-5p		↓	
mmu-miR-148a		↑	↑
mmu-miR-139-5p	↓↓	↓	
mmu-miR-132	↑	↑	
mmu-miR-539	↓		
mmu-miR-125a-5p	↑↑	↑	↓
mmu-miR-130b		↑	

* miRNAs with Nanostring counts that passed the minimum intensity filter and have >2-fold differences among any two-way comparison. Arrows indicate fold changes that are >2-fold up (↑), >5-fold up (↑↑), >2-fold down (↓) or >5-fold down (↓↓). miRNAs are ordered by rows according to expression in C57BL/6 naïve CD4^+^ T cells beginning with highest expression on the top. Exact values of fold changes can be found in Table S4. LAT Y136F indicates LAT Y136F CD4^+^ T cells, B6 HP indicates C57BL/6 CD4^+^ T cells undergoing homeostatic proliferation and B6 H poly indicates C57BL/6 CD4^+^ T cells from *H. polygyrus*-infected mice.

**Figure 4:**
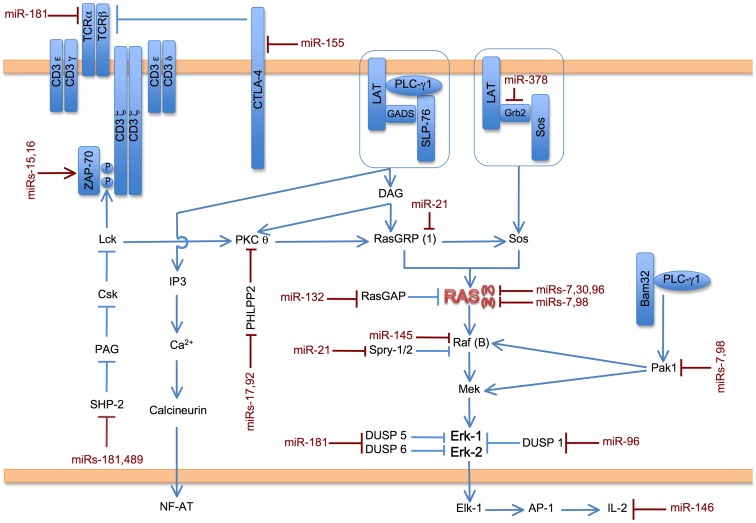
Potential impact of miRs expressed in CD4^+^ T cells on Ras/Erk signaling. In T cells, activation of the multi-subunit TCR (T cell antigen receptor) results in activation of the protein tyrosine kinase ZAP-70. ZAP-70 phosphorylates LAT (Linker for activation of T cells), which acts as a nucleating center for complexes of LAT/Gads/SLP-76/PLC-γ1 and LAT/Grb2/Sos. Enzymatic action from these complexes leads to activation of Ras and downstream activation of Raf, Mek and Erk. Erk activation then leads to T cell proliferation and, in some pathological cases, lymphoproliferative disease. miRNAs that are expressed in this study of CD4^+^ T cells (see 86 miRNAs listed in Table S1) that have known targets in the TCR/ZAP-70/LAT/Ras/Erk pathway are shown in red juxtaposed to their targets. Descriptions of individual targets and references can be found in Table S6. If a given miRNA has been shown to target a particular isoform of a protein, the isoform is indicated in parentheses.

**Figure 5:**
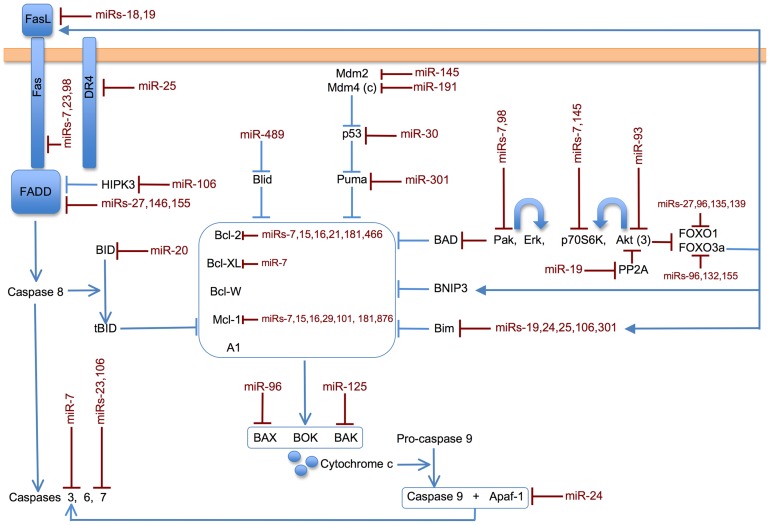
Potential impact of miRs expressed in CD4^+^ T cells on apoptotic pathways. Apoptosis can be initiated from cell surface receptors, such as Fas, utilizing the “extrinsic” pathway. Various forms of stress can initiate apoptosis via the “intrinsic” pathway, which regulates the balance of pro-survival/anti-apoptotic molecules (e.g. Bcl-2, Bcl-XL and others pictured in the central box) and pro-apoptotic molecules (multiple examples are pictured around the central box). miRNAs that are expressed in this study of CD4^+^ T cells (see 86 miRNAs listed in Table S1) that have known targets in the apoptotic pathways shown are printed in red juxtaposed to their targets. Descriptions of individual targets and references can be found in Table S6. If a given miRNA has been shown to target a particular isoform of a protein, the isoform is indicated in parentheses.

By examining miRNA expression in three different proliferative states, we found that miR-21 and miR-146a are highly upregulated in all three settings. In addition we can compose a list of other miRNAs that are differentially regulated more than 5-fold among various combinations of the three proliferative states: miR-96, miR-125a, miR-139, miR-148a, miR-155, miR-181a, miR-361, miR-466a/b and miR669f. The most highly expressed miRNAs that are regulated in LAT Y136F lymphoproliferative disease compared to the other proliferative settings are miR-669f, miR-155 and miR-466a/b. One way of discerning which of these miRNAs merit further study is to survey their known targets and known roles of action in other systems by examination of the literature. To this end, we have reviewed publications describing targets of the 86 miRNAs listed in Table S1 that were expressed above a minimum threshold. Furthermore, we have organized the targets among pathways which include MAPK signaling (including a separate analysis for Erk signaling, Figure 4 and Table S6, and Jnk and classical p38 signaling, Figure S3 and Table S6), apoptosis (Figure 5 and Table S6), PI3K signaling (Figure S4 and Table S6), NFκB signaling (Figure S5 and Table S6), transcriptional activation and cytoskeletal regulation (Table S6).

We have previously shown the importance of the Ras/Erk signaling pathway downstream of the TCR for lymphoproliferative disease in LAT Y136F mice [6], [7]. Examination of miRNAs that are differentially expressed among CD4^+^ T cells with targets in the Ras/Erk pathway reveals several miRNAs that may contribute to CD4^+^ T cell proliferation. Figure 4 depicts TCR signal transduction, which originates from the multi-subunit TCR. Activation of the TCR results in the activation of several protein tyrosine kinases including ZAP-70, which phosphorylates LAT. LAT acts as a nucleating center for other signaling proteins, some of which are depicted in Figure 4. Among them, recruitment of PLC-γ1 and Sos lead to the activation of Ras. In the classic cascade, Ras activation then leads to Raf, Mek and Erk activation [22]. miR-21, which is highly overexpressed in all three proliferative systems compared to wild type naïve CD4^+^ T cells, can upregulate Erk activity through repression of its targets Sprouty 1 and 2 [23], which would lead to upregulation of Raf, Mek and Erk. However, miR-21 overexpression could also cause downregulation of the Ras/Erk pathway by targeting RasGRP [24]. miR-181a, which is underexpressed in lymphoproliferative disease and in homeostatic proliferation, has multiple targets in the Ras/Erk pathway. miR-181a could potentially down-regulate TCR signaling by targeting TCRα [25]. So underexpression of miR-181a could lead to increased signaling. However, the opposite effect would be predicted based on other miR-181a targets [26]. Two other miRNAs highlighted as overexpressed in this study, miR-155 and miR-96, could contribute to Erk signaling and T cell proliferation by targeting the inhibitory signaling molecules CTLA-4 and DUSP 1, respectively [27], [28].

We have previously shown that LAT Y136F T cells have defective restimulation-induced apoptosis [4] and we wanted to analyze apoptotic pathways since the balance of proliferation and apoptosis determines T cell expansion. Figure 5 shows miRNAs with known targets in the extrinsic and intrinsic pathways of apoptosis. The extrinsic pathway is triggered by activation of cell surface receptors, such as Fas. The intrinsic pathway is triggered by cell stress and results in changes in mitochondrial integrity. The balance of proapoptotic (e.g. Bax, Bak, Bad and Bim) and antiapoptotic (e.g. Bcl-2, Bcl-Xl, Mcl-1 and A1) Bcl-2 family members contributes to cytochrome c release from mitochondria in the intrinsic pathway. Both pathways utilize caspases to effect protein degradation [29]. miR-181a, which is underexpressed in lymphoproliferative disease and in homeostatic proliferation, targets Bcl-2 [25], which is anti-apoptotic. Therefore underexpression of miR-181a could lead to lower levels of apoptosis, which could lead to an overall lymphoproliferative outcome. Overexpression of two miRs highlighted in this study could also lead to lower levels of apoptosis: miR-155 by targeting FADD [30] and miR-96 by targeting the pro-apoptotic protein Bax [28].

We have also shown hyper-activation of Jnk and p38 in LAT Y136F T cells [6]. By performing analyses similar to those presented above, we can deduce that the following miRNAs could have an effect on lymphoproliferation via the Jnk and classical p38 signaling pathways (see Figure S3 and Table S6). miR-181a underexpression could increase signaling by targeting TCRα and CD69 [25] and miR-96 overexpression could increase signaling by targeting DUSP 1 [28]. In the PI3K pathway (Figure S4 and Table S6), miR-181a underexpression could result in altered signaling by targeting SirT1 [31]. miR-21 and miR-148a overexpression could result in increased signaling by targeting PTEN [32], [33] and miR-96 and miR-155 overexpression could increase signaling by targeting FOXO3a [34], [35]. In the NFkB pathway (Figure S5 and Table S6), miR155 overexpression could lead to increased signaling by targeting RIPK1 [30]. Further explanations of functions of these targets can be found in Table S6.

The two miRNAs that were overexpressed in all CD4^+^ proliferating cells studied here compared to wild type naïve CD4^+^ T cells were miR-21 and miR-146a. miR-21 has been shown both to be involved in proliferation in autoimmune disease (reviewed in [17]) and in many other settings (e.g. [32], [36], [37], [38]). The miR-21 knockout mouse model showed an increase in Th1 polarization, which is consistent with our observation of miR-21 upregulation in Th2-polarized LAT Y136F CD4^+^ T cells [39]. miR-21 knockdown has also been shown to decrease proliferation in a number of cancer cell types (e.g. [32], [36], [37], [40], [41]). Moreover, by using seed-targeted silencing miR-21 LNAs (locked nucleic acids) in vivo in lupus-prone B6.Sle123 mice, autoimmune splenomegaly was reduced [42]. Therefore accumulating evidence shows that miR-21 is involved in proliferation.

miR-146a has previously been shown to be involved in various states of immune dysregulation. Several studies from the Baltimore lab [16], [43], [44], including characterization of the miR-146a knockout mouse, have shown a role for miR-146a in inflammation and in myeloid malignancies. Multiple studies have also shown a correlation of miR-146a expression with autoimmune disease [45], [46], [47]. miR-146a-deficient Treg cells have been shown to be defective in controlling Th1 immunopathology and in maintaining immune homeostasis [44]. In Jurkat T cells, miR-146a controls IL-2 expression and activation-induced cell death [48]. Recently, Yang et al. have shown that miR-146a controls the resolution of T cell responses in mice [49]. These data regarding resolution of a proliferative response contrast with our hypothesis that miR-146a contributes to proliferation, however miRNA effects vary depending on cell type and environment. This accentuates the need for documentation of miRNA expression in multiple cell types and environments.

In summary, we compared miRNA expression in hyper-proliferative LAT Y136F CD4^+^ T cells to expression in CD4^+^ T cells from mice undergoing homeostatic T cell proliferation and from mice infected with *H. polygyrus*. The overall pattern of miRNA expression in LAT Y136F T cells was similar to that of the other two proliferative systems. The miRNA signature of all three proliferative systems was similar to that of wild type memory CD4^+^ T cells, with *H.polygyrus* infection showing the most similarity to wild type memory T cells. All three proliferative groups and wild type memory T cells were most different from wild type naïve CD4^+^ T cells. However, individual miRNAs showed some interesting patterns of expression. For example, miR-21 and miR-146a were overexpressed compared to wild type naïve CD4^+^ T cells in all three proliferative settings. In contrast, when miRNA expression in LAT Y136F CD4^+^ T cells was compared to miRNA expression in the other two proliferative settings, miR-669f, miR-155 and miR-466a/b were preferentially upregulated when using wild type naïve CD4^+^ T cells as a basis for comparison. Future experiments will directly assess the roles of these miRNAs in T cell proliferation.

## Supporting Information

Figure S1 **miRNA levels from the 21 miRNAs most highly expressed in C57BL/6 naïve CD4^+^ T cells.** miRNA expression levels were determined using the nCounter® mouse miRNA expression assay kit and nCounter® analysis system (Nanostring Technologies). The 21 miRNAs most highly expressed in C57BL/6 naïve CD4^+^ T cells (miR-720 through miR-106b from Table S7 with dead miRNAs removed) are depicted.
(PDF)

Figure S2 **Unsupervised cluster analysis of miRNA expression in T cells undergoing proliferation during helminth infection, homeostatic proliferation, and lymphoproliferative disease.** 19 miRNAs (in rows) showing the highest variance across the three proliferative states are clustered. Probes were median-centered and clustering was done using Euclidean distance and average linkage. Red indicates higher expression, green indicates lower expression, with respect to median.
(PDF)

Figure S3 **Potential impact of miRs expressed in CD4^+^ T cells on Jnk and classical p38 signaling pathways.** Signaling through the TCR (as depicted in Figure 4) results in activation of Pak via activation of the adapter proteins LAT, Bam32 and GIT. Pak activation results in activation of the MAPK Jnk and p38 pathways. Downstream of Jnk and p38, transcription factors that regulate T cell proliferation are activated. miRNAs that are expressed in this study (see 86 miRNAs listed in Table S1) that have known targets in the Jnk/classical p38 pathways are shown in red juxtaposed to their targets. Descriptions of individual targets and references can be found in Table S6. If a given miRNA has been shown to target a particular isoform of a protein, the isoform is indicated in parentheses.
(PDF)

Figure S4 **Potential impact of miRs expressed in CD4^+^ T cells on PI3kinase signaling.** In T cells, PI3K activation results in activation of Akt (aka PKB) and Ras. In general, Akt acts to promote cell survival by downregulating pro-apoptotic molecules such as Bim. Akt also regulates the mTOR pathway, which also modulates cell survival. miRNAs that are expressed in this study of CD4^+^ T cells (see 86 miRNAs listed in Table S1) that have known targets in the PI3K pathway are shown in red juxtaposed to their targets. Descriptions of individual targets and references can be found in Table S6. If a given miRNA has been shown to target a particular isoform of a protein, the isoform is indicated in parentheses.
(PDF)

Figure S5 **Potential impact of miRs expressed in CD4^+^ T cells on NFκB signaling.** Activation of PKCθ downstream of LAT in T cells leads to activation of the CARMA/Bcl-10/Malt1 complex and downstream activation of NFκB via degradation of IκBα. NFκB can then translocate to the nucleus and promote transcription of genes involved in T cell proliferation. miRNAs that are expressed in this study of CD4^+^ T cells (see 86 miRNAs listed in Table S1) that have known targets in the NFκB pathway are shown in red juxtaposed to their targets. Descriptions of individual targets and references can be found in Table S6. If a given miRNA has been shown to target a particular isoform of a protein, the isoform is indicated in parentheses.
(PDF)

Table S1 Fold changes of miRNAs relative to C57BL/6 naïve CD4^+^ T cells*. *Fold changes for miRNAs with Nanostring counts that passed the minimum intensity filter. miRNAs are ordered by rows according to expression in C57BL/6 naïve CD4^+^ T cells beginning with highest expression on the top. LAT Y136F indicates LAT Y136F CD4^+^ T cells, B6 HP indicates C57BL/6 CD4^+^ T cells undergoing homeostatic proliferation, B6 H poly indicates C57BL/6 CD4^+^ T cells from *H. polygyrus*-infected mice and B6 memory indicates C57BL/6 CD4^+^ T cells that are also CD44^hi^CD62L^lo^. B6 naïve CD4^+^ T cells are C57BL/6 CD4^+^ T cells that are also CD44^lo^CD62L^hi^.
(PDF)

Table S2 Fold changes of miRNAs relative to C57BL/6 memory CD4^+^ T cells*. *Fold changes for miRNAs with Nanostring counts that passed the minimum intensity filter. miRNAs are ordered by rows according to expression in C57BL/6 naïve CD4^+^ T cells beginning with highest expression on the top. LAT Y136F indicates LAT Y136F CD4^+^ T cells, B6 HP indicates C57BL/6 CD4^+^ T cells undergoing homeostatic proliferation, B6 H poly indicates C57BL/6 CD4^+^ T cells from *H. polygyrus*-infected mice and B6 memory indicates C57BL/6 CD4^+^ T cells that are also CD44^hi^CD62L^lo^.
(PDF)

Table S3 miRNAs levels as determined by Taqman analysis*. *Fold changes were compared among groups using 2 way anova with Fcrit for a = 0.05 and fold changes were compared within groups using 1 way anova with Fcrit for a = 0.05. KI denotes LAT Y136F CD4^+^ T cells and naïve and memory CD4^+^ T cells are from C57BL/6 (wild type) mice.
(PDF)

Table S4 Fold changes of miRNAs among three proliferative model systems*. *Fold differences of miRNAs with Nanostring counts that passed the minimum intensity filter. miRNAs are ordered by rows according to expression in C57BL/6 naïve CD4^+^ T cells beginning with highest expression on the top. LAT Y136F indicates LAT Y136F CD4^+^ T cells, B6 HP indicates C57BL/6 CD4^+^ T cells undergoing homeostatic proliferation and B6 H poly indicates C57BL/6 CD4^+^ T cells from *H. polygyrus*-infected mice.
(PDF)

Table S5 Log2 expression levels of miRNAs*. *Log2 transformation of Nanostring counts for the 86 miRNAs which passed the minimum intensity filter and for which there were >2 fold differences between any of the samples. The order of rows corresponds to the clustering depicted in Figure 3. B6 naïve T cells are C57BL/6 CD4^+^ T cells that are also CD44^lo^CD62L^hi^ and B6 memory T cells are C57BL/6 CD4^+^ T cells that are also CD44^hi^CD62L^lo^. LAT Y136F indicates LAT Y136F CD4^+^ T cells, B6 HP indicates C57BL/6 CD4^+^ T cells undergoing homeostatic proliferation and B6 H poly indicates C57BL/6 CD4^+^ T cells from *H. polygyrus*-infected mice.
(PDF)

Table S6 **Targets of miRNAs regulated in CD4^+^ T cells (targets of miRNAs shown in italics may not be direct).** Pak1 and Pak2 kinases are equally involved in MAPK transduction and cytoskeletal rearrangement. Pak1 and Pak2, and their upstream regulators such as Vav2, Tiam1, Rac1, Cdc42, and RhoGDI are described in the MAPK table.
(PDF)

Table S7 Nanostring analysis counts of microRNAs from C57BL/6 naïve CD4^+^ T Cells*. *miRNAs include all miRNAs reported from Nanostring Technologies including “dead” miRNAs.
(PDF)

References S1 **Supplemental Biography.**
(PDF)
