# Supplementary figures and images for: miRNA Signature of Mouse Helper T Cell Hyper-Proliferation
Source: PLoS One. 2013 Jun 25;8(6):e66709. doi: 10.1371/journal.pone.0066709 (PMC3692518; doi:10.1371/journal.pone.0066709)

Figure S1.

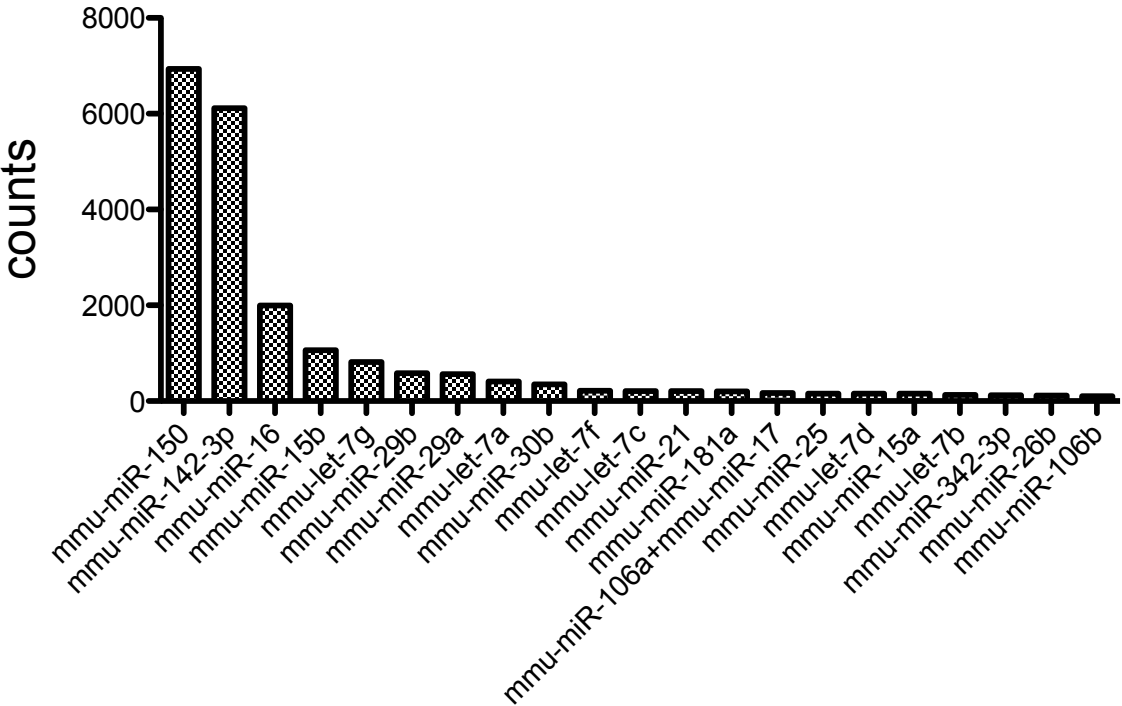

Supplement: Figure S1 — miRNA levels from the 21 miRNAs most highly expressed in C57BL/6 naïve CD4+ T cells. miRNA expression levels were determined using the nCounter® mouse miRNA expression assay kit and nCounter® analysis system (Nanostring Technologies). The 21 miRNAs most highly expressed in C57BL/6 naïve CD4+ T cells (miR-720 through miR-106b from Table S7 with dead miRNAs removed) are depicted. (PDF) [file pone.0066709.s001.pdf]

Figure S2.

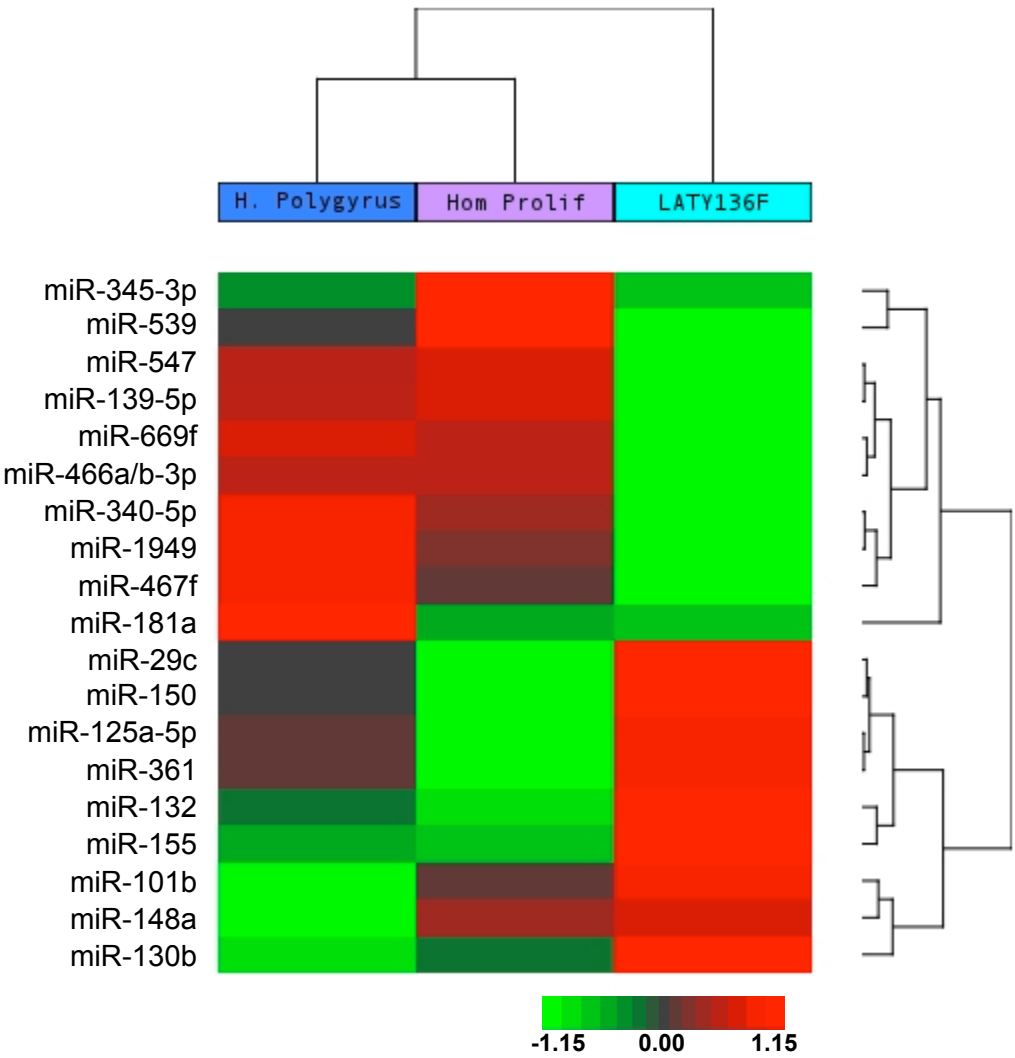

Supplement: Figure S2 — Unsupervised cluster analysis of miRNA expression in T cells undergoing proliferation during helminth infection, homeostatic proliferation, and lymphoproliferative disease. 19 miRNAs (in rows) showing the highest variance across the three proliferative states are clustered. Probes were median-centered and clustering was done using Euclidean distance and average linkage. Red indicates higher expression, green indicates lower expression, with respect to median. (PDF) [file pone.0066709.s002.pdf]

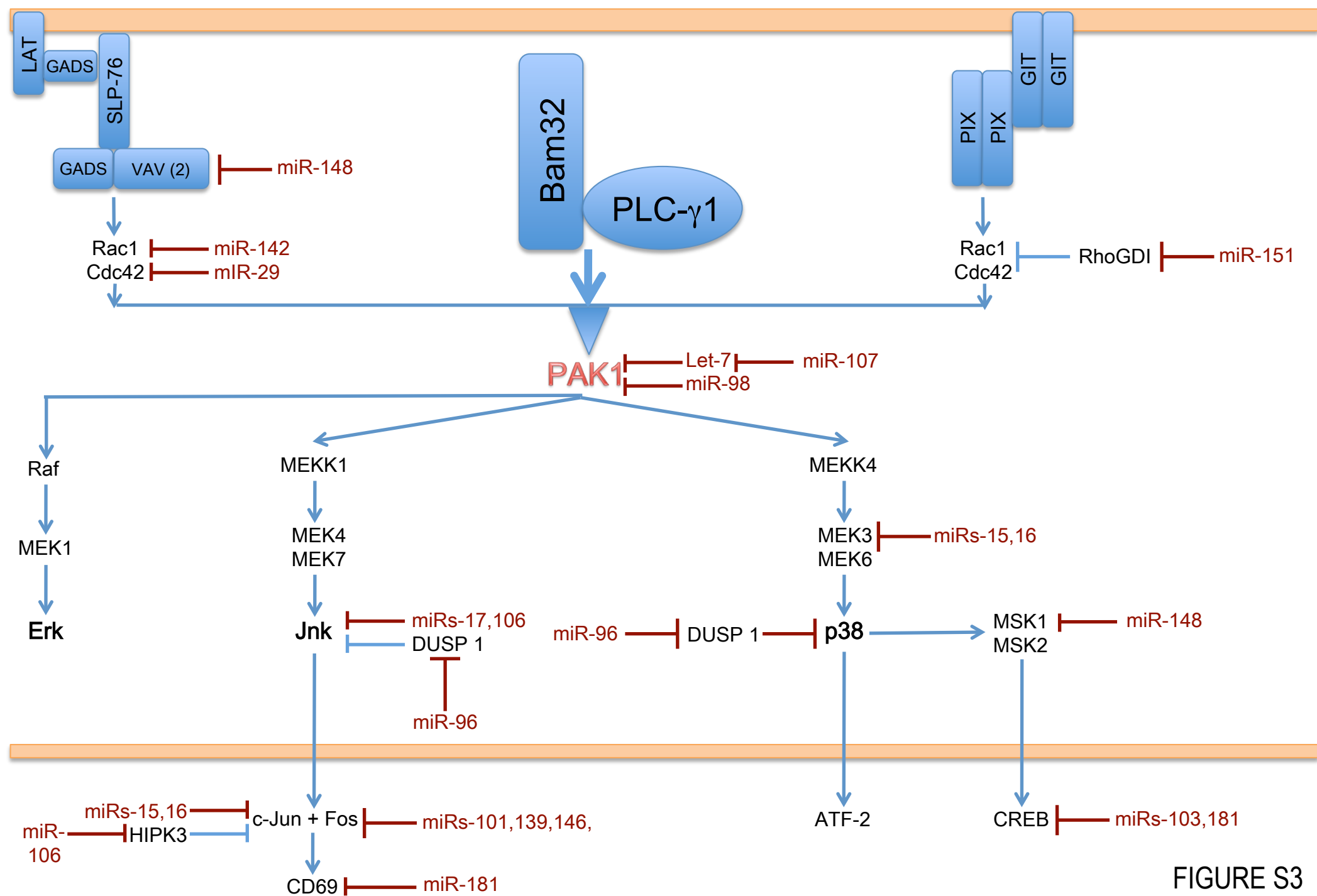

FIGURE S3

Supplement: Figure S3 — Potential impact of miRs expressed in CD4+ T cells on Jnk and classical p38 signaling pathways. Signaling through the TCR (as depicted in Figure 4) results in activation of Pak via activation of the adapter proteins LAT, Bam32 and GIT. Pak activation results in activation of the MAPK Jnk and p38 pathways. Downstream of Jnk and p38, transcription factors that regulate T cell proliferation are activated. miRNAs that are expressed in this study (see 86 miRNAs listed in Table S1) that have known targets in the Jnk/classical p38 pathways are shown in red juxtaposed to their targets. Descriptions of individual targets and references can be found in Table S6. If a given miRNA has been shown to target a particular isoform of a protein, the isoform is indicated in parentheses. (PDF) [file pone.0066709.s003.pdf]

FIGURE S4

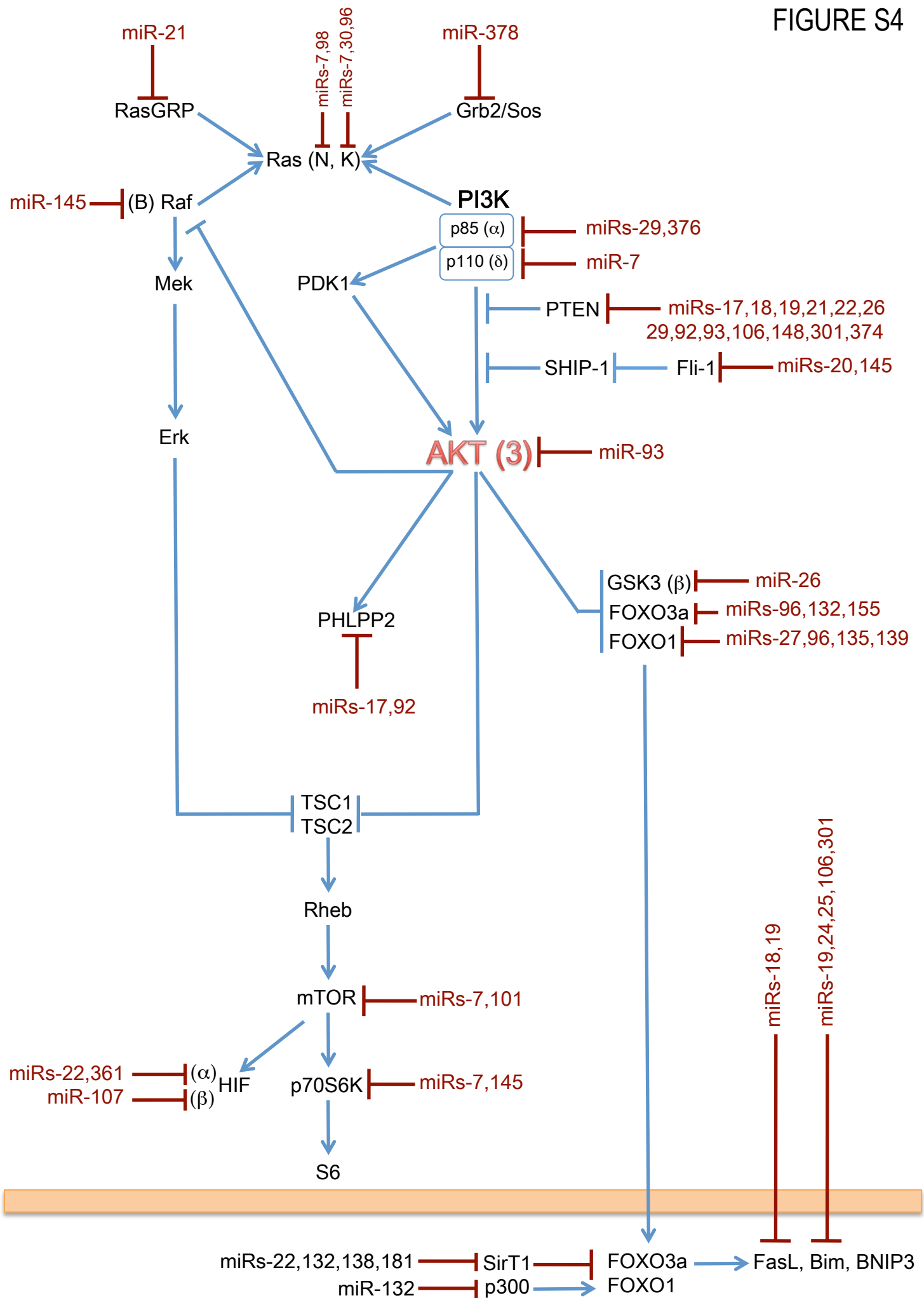

Supplement: Figure S4 — Potential impact of miRs expressed in CD4+ T cells on PI3kinase signaling. In T cells, PI3K activation results in activation of Akt (aka PKB) and Ras. In general, Akt acts to promote cell survival by downregulating pro-apoptotic molecules such as Bim. Akt also regulates the mTOR pathway, which also modulates cell survival. miRNAs that are expressed in this study of CD4+ T cells (see 86 miRNAs listed in Table S1) that have known targets in the PI3K pathway are shown in red juxtaposed to their targets. Descriptions of individual targets and references can be found in Table S6. If a given miRNA has been shown to target a particular isoform of a protein, the isoform is indicated in parentheses. (PDF) [file pone.0066709.s004.pdf]

FIGURE S5

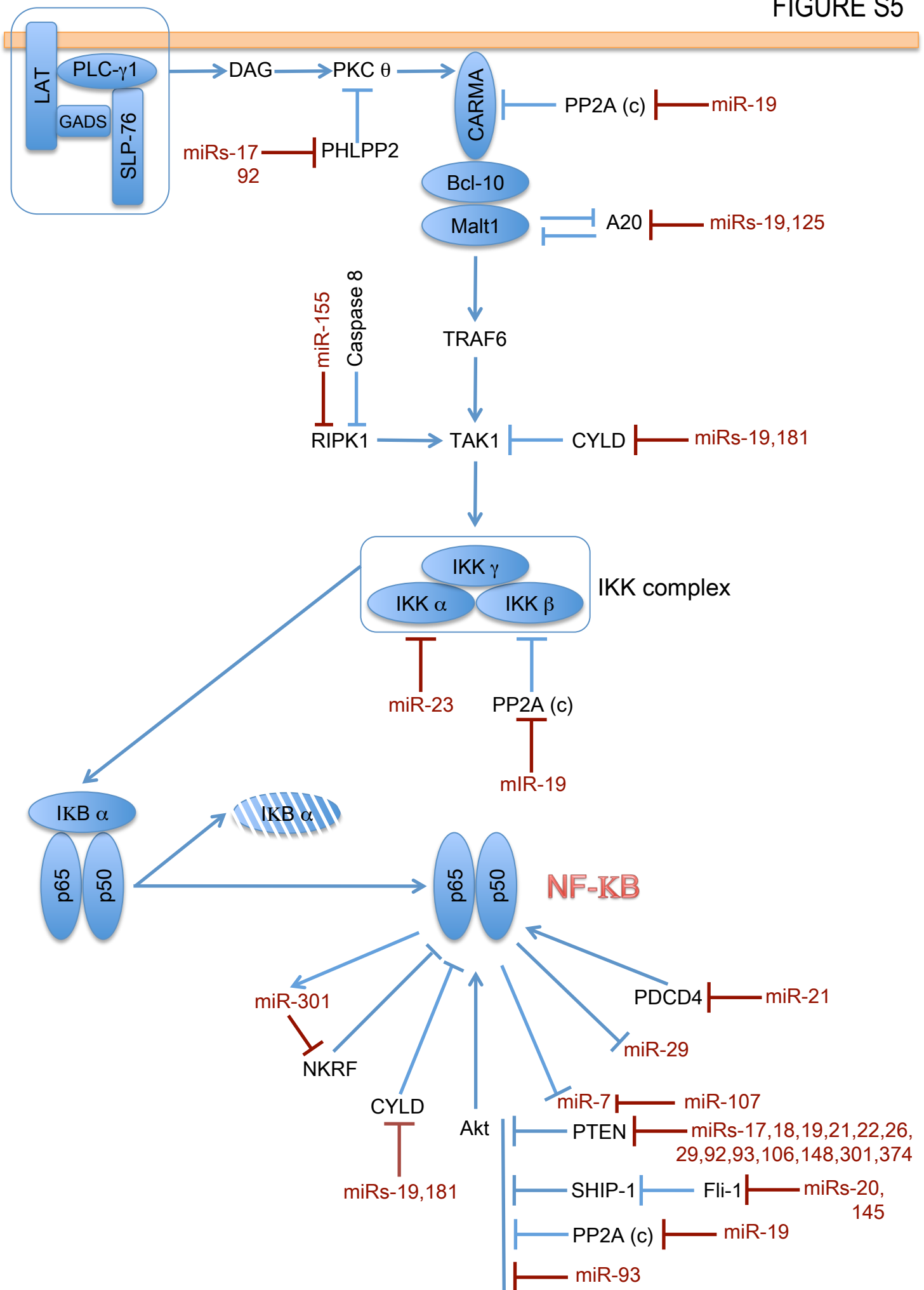

Supplement: Figure S5 — Potential impact of miRs expressed in CD4+ T cells on NFκB signaling. Activation of PKCθ downstream of LAT in T cells leads to activation of the CARMA/Bcl-10/Malt1 complex and downstream activation of NFκB via degradation of IκBα. NFκB can then translocate to the nucleus and promote transcription of genes involved in T cell proliferation. miRNAs that are expressed in this study of CD4+ T cells (see 86 miRNAs listed in Table S1) that have known targets in the NFκB pathway are shown in red juxtaposed to their targets. Descriptions of individual targets and references can be found in Table S6. If a given miRNA has been shown to target a particular isoform of a protein, the isoform is indicated in parentheses. (PDF) [file pone.0066709.s005.pdf]
