# Supplementary material for: miRNA Signature of Mouse Helper T Cell Hyper-Proliferation
Source: PLoS One. 2013 Jun 25;8(6):e66709. doi: 10.1371/journal.pone.0066709 (PMC3692518; doi:10.1371/journal.pone.0066709)
Supplement: Table S1 — Fold changes of miRNAs relative to C57BL/6 naïve CD4+ T cells*. *Fold changes for miRNAs with Nanostring counts that passed the minimum intensity filter. miRNAs are ordered by rows according to expression in C57BL/6 naïve CD4+ T cells beginning with highest expression on the top. LAT Y136F indicates LAT Y136F CD4+ T cells, B6 HP indicates C57BL/6 CD4+ T cells undergoing homeostatic proliferation, B6 H poly indicates C57BL/6 CD4+ T cells from H. polygyrus-infected mice and B6 memory indicates C57BL/6 CD4+ T cells that are also CD44hiCD62Llo. B6 naïve CD4+ T cells are C57BL/6 CD4+ T cells that are also CD44loCD62Lhi. (PDF) [file pone.0066709.s006.pdf]

Table S1. Fold changes of miRNAs relative to C57BL/6 naïve CD4+ T cells*

miRNA	LAT Y136F vs B6 naïve	HP vs B6 naïve	H poly vs B6 naïve	B6 memory vs B6 naïve
**mmu-miR-150**	-1.35482	-2.77907	-1.96482	-2.97647
**mmu-miR-142-3p**	-1.38521	-1.82792	-1.23576	-1.38992
**mmu-miR-16**	-1.337	-1.29199	-1.13179	1.25571
**mmu-miR-15b**	-2.12532	-1.72511	-1.31342	1.36424
**mmu-let-7g**	-1.41584	-1.34702	-1.03053	1.05342
**mmu-miR-29b**	1.50905	-1.11697	1.42198	1.43574
**mmu-miR-29a**	-1.22558	-1.79196	1.01611	-1.15102
**mmu-let-7a**	1.10082	-1.22721	1.15579	1.15309
**mmu-miR-30b**	-1.31424	-2.46629	-1.31385	-1.42718
**mmu-let-7f**	-1.19744	-2.07268	-1.20189	-1.24318
**mmu-miR-21**	23.1061	19.2715	13.4264	15.2338
**mmu-let-7c**	-2.10025	-4.16313	-1.75139	-1.55021
**mmu-miR-181a**	-32.7758	-26.3909	-1.91798	-1.87395
**mmu-miR-106+17**	1.38487	1.45381	1.1702	1.20891
**mmu-miR-25**	1.02886	1.17207	1.06452	-1.02498
**mmu-miR-15a**	2.42214	2.78146	2.04402	1.86466
**mmu-let-7d**	1.11993	1.26657	1.28413	1.2849
**mmu-let-7b**	-2.24376	-2.69813	-1.48967	-2.5457
**mmu-miR-342-3p**	-1.39412	-2.33432	1.26386	-1.42557
**mmu-miR-26b**	-1.73215	-2.61445	-1.27622	-1.19133
**mmu-miR-106b**	1.05965	1.26879	1.00144	1.22214
**mmu-miR-19a**	1.08367	1.11545	-1.46489	-1.20559
**mmu-miR-669f**	-7.15291	-2.31585	-2.09166	-1.83523
**mmu-miR-30d**	-1.28384	-1.29986	-1.28178	-1.10366
**mmu-miR-20a/b**	1.00278	-1.36273	-1.16651	-1.1749
**mmu-miR-151-3p**	-1.61619	-2.38596	-2.4954	-3.49049
**mmu-miR-297c**	-1.10733	1.69984	-1.18311	-1.59819
**mmu-miR-29c**	2.57157	1.22935	1.82221	1.55141
**mmu-miR-30e**	-1.74793	-1.59263	-1.36327	-1.01568
**mmu-miR-151-5p**	-1.26627	-2.12444	-1.98393	-2.69316
**mmu-miR-19b**	1.19224	1.14125	-1.20405	-1.30645
**mmu-miR-155**	5.25492	2.25172	2.45374	5.0861
**mmu-miR-467f**	-4.79018	-2.80482	-1.86695	-1.98231
**mmu-miR-378**	-2.33777	-2.66257	-2.27912	-1.45057
**mmu-miR-466a/b-3p**	-9.12889	-2.90585	-2.84826	-1.55391
**mmu-miR-146a**	24.2971	25.6629	20.4781	23.4727
**mmu-miR-145**	-1.62672	1.01481	-2.32609	-1.32428
**mmu-miR-10a**	-2.2669	-2.13941	-2.26236	-1.59851
**mmu-miR-191**	1.59708	1.14734	1.37818	1.32273
**mmu-miR-27a**	3.16166	2.77751	2.84212	2.56662

**mmu-miR-140**	-1.24351	-1.04574	1.05318	1.52832
**mmu-miR-23b**	1.95055	1.00397	1.63841	1.2669
**mmu-miR-361**	1.531	-3.31282	-1.24894	-1.31239
**mmu-miR-22**	2.84567	3.42193	3.2948	2.28617
**mmu-miR-26a**	-1.24725	1.09288	1.00312	-1.06118
**mmu-miR-2183**	-1.41908	1.23743	1.03275	-1.24318
**mmu-miR-423-5p**	-1.50528	-2.53896	-1.16207	-1.05653
**mmu-miR-30c**	-1.55242	-1.97574	-1.61397	-1.62547
**mmu-miR-466g**	-1.37759	-1.15038	-1.00728	-1.07968
**mmu-miR-547**	-2.19141	-1.01529	-1.1281	-1.46513
**mmu-miR-338-5p**	-1.09571	-1.23283	-2.01457	-1.24344
**mmu-let-7i**	2.38494	1.79639	1.48916	1.48675
**mmu-miR-883b-3p**	-1.61774	1.23373	-1.55872	-2.56918
**mmu-miR-103**	1.54537	1.23373	1.92498	1.32318
**mmu-miR-374**	1.86459	1.58279	1.29139	-1.33501
**mmu-miR-30a**	-1.77841	-1.62739	-1.4078	-1.5137
**mmu-miR-1949**	-3.94195	-2.01633	-1.53794	-1.01723
**mmu-miR-345-3p**	-1.30997	1.83868	-1.1113	-1.40569
**mmu-miR-101b**	1.25924	-1.08922	-1.7799	-1.63525
**mmu-miR-340-5p**	-2.50415	-1.39457	-1.13921	-1.49196
**mmu-miR-544**	-1.3486	1.19553	-2.05098	-1.42084
**mmu-miR-423-3p**	-1.1312	-1.04547	-1.36693	-1.65682
**mmu-miR-200b**	-1.16844	1.43455	1.3169	-1.24305
**mmu-miR-98**	1.02666	1.30411	1.17036	1.13376
**mmu-miR-148b**	-1.16909	-1.49369	-1.06794	1.04572
**mmu-miR-669i**	-1.39142	-1.30735	-1.31503	-1.46221
**mmu-miR-148a**	10.0523	7.75142	2.83359	2.79487
**mmu-miR-484**	1.71067	1.48703	1.43015	1.3407
**mmu-miR-139-5p**	-3.75726	1.48703	1.23523	1.07234
**mmu-miR-301a**	1.96707	1.79255	1.53541	1.25652
**mmu-miR-135b**	-1.46079	-1.30763	1.01392	1.01922
**mmu-miR-376a**	1.87078	1.78462	1.79457	1.23327
**mmu-miR-489**	1.00398	1.65739	-1.60191	-1.33186
**mmu-miR-876-3p**	1.00398	1.27502	-1.83132	-1.33186
**mmu-miR-132**	2.5096	-1.5686	-1.06794	1.71637
**mmu-miR-23a**	3.46787	3.95229	4.68059	3.75413
**mmu-miR-539**	1.09542	2.2949	1.55997	1.23327
**mmu-miR-93**	1.85795	3.00568	1.83948	1.14914
**mmu-miR-125a-5p**	5.9659	1.09304	2.92612	2.70099
**mmu-miR-130b**	2.52676	1.47095	1.08028	1.36086
**mmu-let-7e**	2.36851	1.32339	1.25994	1.54663
**mmu-miR-350**	1.19802	1.27506	1.95029	2.01161
**mmu-miR-24**	3.42255	2.55013	2.04806	2.41342

**mmu-miR-107**	2.48074	2.86833	3.21739	2.61366
**mmu-miR-1902**	4.10795	2.73295	4.84941	2.0696
**mmu-miR-96**	5.67327	7.92467	3.00993	1.83948

*Fold changes for miRNAs with Nanostring counts that passed the minimum intensity filter. miRNAs are ordered by rows according to expression in C57BL/6 naïve $CD4^+$ T cells beginning with highest expression on the top. LAT Y136F indicates LAT Y136F $CD4^+$ T cells, B6 HP indicates C57BL/6 $CD4^+$ T cells undergoing homeostatic proliferation, B6 H poly indicates C57BL/6 $CD4^+$ T cells from *H. polygyrus*-infected mice and B6 memory indicates C57BL/6 $CD4^+$ T cells that are also $CD44^{hi}CD62L^{lo}$. B6 naïve $CD4^+$ T cells are C57BL/6 $CD4^+$ T cells that are also $CD44^{lo}CD62L^{hi}$.
