# Supplementary material for: miRNA Signature of Mouse Helper T Cell Hyper-Proliferation
Source: PLoS One. 2013 Jun 25;8(6):e66709. doi: 10.1371/journal.pone.0066709 (PMC3692518; doi:10.1371/journal.pone.0066709)
Supplement: Table S2 — Fold changes of miRNAs relative to C57BL/6 memory CD4+ T cells*. *Fold changes for miRNAs with Nanostring counts that passed the minimum intensity filter. miRNAs are ordered by rows according to expression in C57BL/6 naïve CD4+ T cells beginning with highest expression on the top. LAT Y136F indicates LAT Y136F CD4+ T cells, B6 HP indicates C57BL/6 CD4+ T cells undergoing homeostatic proliferation, B6 H poly indicates C57BL/6 CD4+ T cells from H. polygyrus-infected mice and B6 memory indicates C57BL/6 CD4+ T cells that are also CD44hiCD62Llo. (PDF) [file pone.0066709.s007.pdf]

Table S2. Fold changes of miRNAs relative to C57BL/6 memory CD4^{+} T cells*

miRNA	LATY136F vs B6 memory	HP vs B6 memory	H poly vs B6 memory
**mmu-miR-150**	2.19695	1.07103	1.51488
**mmu-miR-142-3p**	1.0034	-1.31513	1.12475
**mmu-miR-16**	-1.67888	-1.62237	-1.42119
**mmu-miR-15b**	-2.89945	-2.35347	-1.79183
**mmu-let-7g**	-1.49148	-1.41898	-1.08558
**mmu-miR-29b**	1.05106	-1.60367	-1.00967
**mmu-miR-29a**	-1.06478	-1.55685	1.16956
**mmu-let-7a**	-1.04749	-1.41509	1.00234
**mmu-miR-30b**	1.08594	-1.72808	1.08626
**mmu-let-7f**	1.0382	-1.66724	1.03435
**mmu-miR-21**	1.51677	1.26505	-1.13461
**mmu-let-7c**	-1.35482	-2.68554	-1.12978
**mmu-miR-181a**	-17.4903	-14.0831	-1.0235
**mmu-miR-106a+17**	1.14555	1.20258	-1.03308
**mmu-miR-25**	1.05456	1.20134	1.0911
**mmu-miR-15a**	1.29897	1.49167	1.09619
**mmu-let-7d**	-1.14731	-1.01447	-1.0006
**mmu-let-7b**	1.13457	-1.05988	1.7089
**mmu-miR-342-3p**	1.02256	-1.63746	1.80173
**mmu-miR-26b**	-1.45396	-2.19457	-1.07126
**mmu-miR-106b**	-1.15334	1.03817	-1.22039
**mmu-miR-19a**	1.30646	1.34478	-1.21508
**mmu-miR-669f**	-3.89756	-1.26188	-1.13973
**mmu-miR-30d**	-1.16327	-1.17778	-1.1614
**mmu-miR-20a/b**	1.17816	-1.15987	1.00719
**mmu-miR-151-3p**	2.1597	1.46293	1.39877
**mmu-miR-297c**	1.44329	2.71667	1.35083
**mmu-miR-29c**	1.65757	-1.26198	1.17455
**mmu-miR-30e**	-1.72096	-1.56805	-1.34223
**mmu-miR-151-5p**	2.12685	1.26771	1.35749
**mmu-miR-19b**	1.55759	1.49099	1.08504
**mmu-miR-155**	1.03319	-2.25876	-2.0728
**mmu-miR-467f**	-2.41647	-1.41493	1.06179
**mmu-miR-378**	-1.61162	-1.83553	-1.57119
**mmu-miR-466a/b-3p**	-5.8748	-1.87003	-1.83297
**mmu-miR-146a**	1.03512	1.09331	-1.14623
**mmu-miR-145**	-1.22838	1.34389	-1.7565
**mmu-miR-10a**	-1.41813	-1.33837	-1.41529

**mmu-miR-191**	1.20741	-1.15287	1.04192
**mmu-miR-27a**	1.23184	1.08216	1.10734
**mmu-miR-140**	-1.90049	-1.59822	-1.45115
**mmu-miR-23b**	1.53963	-1.26188	1.29325
**mmu-miR-361**	2.00927	-2.52426	1.05081
**mmu-miR-22**	1.24474	1.4968	1.44119
**mmu-miR-26a**	-1.17534	1.15974	1.0645
**mmu-miR-2183**	-1.14149	1.53836	1.2839
**mmu-miR-423-5p**	-1.42474	-2.40312	-1.0999
**mmu-miR-30c**	1.04706	-1.21548	1.00713
**mmu-miR-466g**	-1.27593	-1.06548	1.07187
**mmu-miR-547**	-1.49571	1.44307	1.29876
**mmu-miR-338-5p**	1.13483	1.00861	-1.62015
**mmu-let-7i**	1.60413	1.20827	1.00162
**mmu-miR-883b-3p**	1.58813	3.16969	1.64827
**mmu-miR-103**	1.16792	-1.0725	1.45481
**mmu-miR-374**	2.48925	2.11304	1.72402
**mmu-miR-30a**	-1.17488	-1.07511	1.07523
**mmu-miR-1949**	-3.8752	-1.98218	-1.5119
**mmu-miR-345-3p**	1.07307	2.58462	1.26491
**mmu-miR-101b**	2.05917	1.50131	-1.08846
**mmu-miR-340-5p**	-1.67843	1.06984	1.30964
**mmu-miR-544**	1.05357	1.69865	-1.4435
**mmu-miR-423-3p**	1.46465	1.58477	1.21208
**mmu-miR-200b**	1.06385	1.78321	1.63697
**mmu-miR-98**	-1.10431	1.15026	1.03228
**mmu-miR-148b**	-1.22254	-1.56198	-1.11677
**mmu-miR-669i**	1.05087	1.11846	1.11192
**mmu-miR-148a**	3.59669	2.77345	1.01385
**mmu-miR-484**	1.27595	1.10914	1.06672
**mmu-miR-139-5p**	-4.02905	1.38671	1.1519
**mmu-miR-301a**	1.5655	1.4266	1.22195
**mmu-miR-135b**	-1.48886	-1.33275	-1.00523
**mmu-miR-376a**	1.51693	1.44707	1.45513
**mmu-miR-489**	1.33716	2.2074	-1.20276
**mmu-miR-876-3p**	1.33716	1.69815	-1.37501
**mmu-miR-132**	1.46216	-2.6923	-1.83298
**mmu-miR-23a**	-1.08255	1.05278	1.24678
**mmu-miR-539**	-1.12584	1.86082	1.26491
**mmu-miR-93**	1.61681	2.61558	1.60074
**mmu-miR-125a-5p**	2.20878	-2.47109	1.08335
**mmu-miR-130b**	1.85674	1.0809	-1.25973
**mmu-let-7e**	1.5314	-1.16869	-1.22755

**mmu-miR-350**	-1.67911	-1.57765	-1.03144
**mmu-miR-24**	1.41813	1.05664	-1.17839
**mmu-miR-107**	-1.05358	1.09744	1.23099
**mmu-miR-1902**	1.98491	1.32052	2.34317
**mmu-miR-96**	3.08417	4.30809	1.63629

*Fold changes for miRNAs with Nanostring counts that passed the minimum intensity filter. miRNAs are ordered by rows according to expression in C57BL/6 naïve $CD4^+$ T cells beginning with highest expression on the top. LAT Y136F indicates LAT Y136F $CD4^+$ T cells, B6 HP indicates C57BL/6 $CD4^+$ T cells undergoing homeostatic proliferation, B6 H poly indicates C57BL/6 $CD4^+$ T cells from *H. polygyrus*-infected mice and B6 memory indicates C57BL/6 $CD4^+$ T cells that are also $CD44^{hi}CD62L^{lo}$.
