# Supplementary material for: miRNA Signature of Mouse Helper T Cell Hyper-Proliferation
Source: PLoS One. 2013 Jun 25;8(6):e66709. doi: 10.1371/journal.pone.0066709 (PMC3692518; doi:10.1371/journal.pone.0066709)
Supplement: Table S3 — miRNAs levels as determined by Taqman analysis*. *Fold changes were compared among groups using 2 way anova with Fcrit for a = 0.05 and fold changes were compared within groups using 1 way anova with Fcrit for a = 0.05. KI denotes LAT Y136F CD4+ T cells and naïve and memory CD4+ T cells are from C57BL/6 (wild type) mice. (PDF) [file pone.0066709.s008.pdf]

Table S3. miRNAs levels as determined by Taqman analysis*

miRNA	Comparison	2 way anova p value	miRNA	Group	1 way anova p value
mmu-miR-21	KI vs naïve	1.81386E-17	mmu-miR-21	KI	0.13715
mmu-miR-21	memory vs naïve	1.07484E-15	mmu-miR-21	memory	0.54437
mmu-miR-21	KI vs memory	0.13994	mmu-miR-21	naïve	0.86858
mmu-miR-146a	KI vs naïve	8.92468E-13	mmu-miR-146a	KI	0.03030
mmu-miR-146a	memory vs naïve	1.29963E-10	mmu-miR-146a	memory	0.85952
mmu-miR-146a	KI vs memory	0.00909	mmu-miR-146a	naïve	0.86858
mmu-miR-148a	KI vs naïve	2.1743E-13	mmu-miR-148a	KI	0.00735
mmu-miR-148a	memory vs naïve	1.80176E-09	mmu-miR-148a	memory	0.01989
mmu-miR-148a	KI vs memory	1.52622E-07	mmu-miR-148a	naïve	0.00396
mmu-miR-181a	KI vs naïve	4.42683E-10	mmu-miR-181a	KI	0.83198
mmu-miR-181a	memory vs naïve	0.02910	mmu-miR-181a	memory	0.14382
mmu-miR-181a	KI vs memory	1.91845E-10	mmu-miR-181a	naïve	0.34122

*Fold changes were compared among groups using 2 way anova with Fcrit for a=0.05 and fold changes were compared within groups using 1 way anova with Fcrit for a=0.05. KI denotes LAT Y136F $CD4^+$ T cells and naïve and memory $CD4^+$ T cells are from C57BL/6 (wild type) mice.
