# Supplementary material for: miRNA Signature of Mouse Helper T Cell Hyper-Proliferation
Source: PLoS One. 2013 Jun 25;8(6):e66709. doi: 10.1371/journal.pone.0066709 (PMC3692518; doi:10.1371/journal.pone.0066709)
Supplement: Table S4 — Fold changes of miRNAs among three proliferative model systems*. *Fold differences of miRNAs with Nanostring counts that passed the minimum intensity filter. miRNAs are ordered by rows according to expression in C57BL/6 naïve CD4+ T cells beginning with highest expression on the top. LAT Y136F indicates LAT Y136F CD4+ T cells, B6 HP indicates C57BL/6 CD4+ T cells undergoing homeostatic proliferation and B6 H poly indicates C57BL/6 CD4+ T cells from H. polygyrus-infected mice. (PDF) [file pone.0066709.s009.pdf]

Table S4. Fold changes of miRNAs among three proliferative model systems*

miRNA	LAT Y136F vs HP	LATY136F vs H poly	HP vs H poly
**mmu-miR-150**	2.05125	1.45025	-1.41441
**mmu-miR-181a**	-1.24194	-17.0887	-13.7597
**mmu-miR-669f**	-3.08868	-3.41972	-1.10718
**mmu-miR-29c**	2.09182	1.41124	-1.48226
**mmu-miR-155**	2.33373	2.1416	-1.08971
**mmu-miR-467f**	-1.70784	-2.56578	-1.50236
**mmu-miR-466a/b-3p**	-3.14156	-3.20508	-1.02022
**mmu-miR-361**	5.07191	1.91212	-2.65251
**mmu-miR-547**	-2.15841	-1.94257	1.11111
**mmu-miR-1949**	-1.95502	-2.56314	-1.31106
**mmu-miR-345-3p**	-2.40861	-1.17877	2.04333
**mmu-miR-101b**	1.37158	2.24132	1.63412
**mmu-miR-340-5p**	-1.79564	-2.19813	-1.22415
**mmu-miR-148a**	1.29683	3.54754	2.73555
**mmu-miR-139-5p**	-5.58714	-4.64108	1.20385
**mmu-miR-132**	3.93657	2.68011	-1.46881
**mmu-miR-539**	-2.09499	-1.42408	1.47111
**mmu-miR-125a-5p**	5.4581	2.03884	-2.67706
**mmu-miR-130b**	1.71777	2.339	1.36164

*Fold differences of miRNAs with Nanostring counts that passed the minimum intensity filter. miRNAs are ordered by rows according to expression in C57BL/6 naïve $CD4^+$ T cells beginning with highest expression on the top. LAT Y136F indicates LAT Y136F $CD4^+$ T cells, B6 HP indicates C57BL/6 $CD4^+$ T cells undergoing homeostatic proliferation and B6 H poly indicates C57BL/6 $CD4^+$ T cells from *H. polygyrus*-infected mice.
