# Supplementary material for: miRNA Signature of Mouse Helper T Cell Hyper-Proliferation
Source: PLoS One. 2013 Jun 25;8(6):e66709. doi: 10.1371/journal.pone.0066709 (PMC3692518; doi:10.1371/journal.pone.0066709)
Supplement: Table S5 — Log2 expression levels of miRNAs*. *Log2 transformation of Nanostring counts for the 86 miRNAs which passed the minimum intensity filter and for which there were >2 fold differences between any of the samples. The order of rows corresponds to the clustering depicted in Figure 3. B6 naïve T cells are C57BL/6 CD4+ T cells that are also CD44loCD62Lhi and B6 memory T cells are C57BL/6 CD4+ T cells that are also CD44hiCD62Llo. LAT Y136F indicates LAT Y136F CD4+ T cells, B6 HP indicates C57BL/6 CD4+ T cells undergoing homeostatic proliferation and B6 H poly indicates C57BL/6 CD4+ T cells from H. polygyrus-infected mice. (PDF) [file pone.0066709.s010.pdf]

Table S5. Log2 expression levels of miRNAs*

miRNA	B6 naive	B6 HP	LATY136F	B6 H poly	B6 memory
**mmu-miR-30d**	6.45154	6.07318	6.09107	6.09339	6.30925
**mmu-miR-378**	5.81609	4.40327	4.59096	4.62761	5.27947
**mmu-miR-10a**	5.50048	4.40327	4.31976	4.32265	4.82375
**mmu-miR-140**	5.33056	5.26604	5.01614	5.40531	5.94251
**mmu-let-7g**	9.67753	9.24776	9.17587	9.63414	9.75261
**mmu-miR-135b**	3.91552	3.52857	3.36877	3.93546	3.94298
**mmu-miR-181a**	7.66683	2.94486	2.63227	6.72724	6.76075
**mmu-miR-15b**	10.0571	9.27041	8.96942	9.66377	10.5052
**mmu-miR-16**	10.9628	10.5932	10.5438	10.7842	11.2913
**mmu-miR-30e**	6.07468	5.40327	5.26903	5.62761	6.05224
**mmu-miR-467f**	5.89118	4.40327	3.6311	4.9905	4.904
**mmu-miR-30a**	4.81609	4.11353	3.9855	4.32265	4.21801
**mmu-miR-669f**	6.54782	5.33628	3.70929	5.48317	5.67186
**mmu-miR-466a/b-3p**	5.65249	4.11353	2.46205	4.14241	5.01659
**mmu-miR-1949**	4.76341	3.75168	2.7845	4.14241	4.73877
**mmu-miR-340-5p**	4.59335	4.11353	3.26903	4.40531	4.01614
**mmu-miR-466g**	5.05311	4.851	4.59096	5.04264	4.94251
**mmu-miR-150**	12.7606	11.286	12.3225	11.7862	11.187
**mmu-miR-151-5p**	6.031	4.94392	5.69042	5.04264	4.6017
**mmu-miR-151-3p**	6.19849	4.94392	5.50589	4.87922	4.39506
**mmu-let-7b**	7.01992	5.58796	5.854	6.44493	5.67186
**mmu-miR-30c**	5.09592	4.11353	4.4614	4.40531	4.39506
**mmu-miR-669i**	4.33056	3.94392	3.854	3.93546	3.78241
**mmu-miR-361**	5.25663	3.52857	5.8711	4.93593	4.86443
**mmu-miR-20a/b**	6.43496	5.98846	6.43896	6.21276	6.20242
**mmu-miR-342-3p**	6.97441	5.75141	6.49506	7.31225	6.46287
**mmu-miR-29a**	9.14308	8.30154	8.84962	9.16614	8.94017
**mmu-miR-142-3p**	12.579	11.7088	12.1089	12.2736	12.104
**mmu-miR-30b**	8.45574	7.1534	8.06151	8.06194	7.94257
**mmu-let-7f**	7.77683	6.72533	7.51688	7.51152	7.46279
**mmu-let-7c**	7.7021	5.64443	6.63154	6.8936	7.06964
**mmu-miR-148b**	4.33056	3.75168	4.10518	4.23573	4.39506
**mmu-miR-26b**	6.85412	5.46761	6.06156	6.50224	6.60155
**mmu-miR-423-5p**	5.09592	3.75168	4.50589	4.87922	5.01659
**mmu-miR-139-5p**	4.17871	4.75114	2.26903	4.48349	4.27947
**mmu-miR-2183**	5.09592	5.40327	4.59096	5.14241	4.78188
**mmu-miR-547**	5.05311	5.03122	3.92125	4.87922	4.50208
**mmu-miR-26a**	5.13791	5.26604	4.81916	5.14241	5.05224
**mmu-miR-883b-3p**	4.96301	5.26604	4.26903	4.32265	3.6017

**mmu-miR-297c**	6.17852	6.94392	6.03144	5.93593	5.50208
**mmu-miR-345-3p**	4.70929	5.58796	4.31976	4.55704	4.21801
**mmu-miR-93**	3.81558	5.40327	4.70929	4.69488	4.01614
**mmu-miR-25**	7.28475	7.51381	7.3258	7.37495	7.24916
**mmu-miR-539**	3.91552	5.11395	4.04701	4.55704	4.21801
**mmu-miR-98**	4.46793	4.851	4.50589	4.69488	4.64904
**mmu-miR-200b**	4.59335	5.11395	4.36877	4.9905	4.27947
**mmu-miR-106b**	6.70901	7.05246	6.7926	6.71108	6.99842
**mmu-miR-19b**	5.96278	6.1534	6.21645	5.69488	5.57713
**mmu-miR-101b**	4.65249	4.5292	4.98504	3.82069	3.94298
**mmu-miR-423-3p**	4.59335	4.5292	4.41549	4.14241	3.86493
**mmu-miR-489**	3.91552	4.64443	3.92125	3.23573	3.50208
**mmu-miR-876-3p**	3.91552	4.26604	3.92125	3.04264	3.50208
**mmu-miR-19a**	6.59365	6.75128	6.70957	6.04286	6.32391
**mmu-miR-145**	5.62322	5.64443	4.92125	4.40531	5.21801
**mmu-miR-544**	4.59335	4.851	4.16189	3.55704	4.08661
**mmu-miR-338-5p**	5.05311	4.75114	4.92125	4.04264	4.73877
**mmu-miR-130b**	3.70929	4.26604	5.04658	3.82069	4.15381
**mmu-let-7e**	3.70929	4.11353	4.95327	4.04264	4.33842
**mmu-miR-155**	5.96278	7.13381	8.35645	7.25776	8.30934
**mmu-miR-125a-5p**	3.81558	3.94392	6.39232	5.36457	5.24907
**mmu-miR-191**	5.50048	5.69877	6.17592	5.96324	5.904
**mmu-miR-29c**	6.13791	6.4358	7.50056	7.0036	6.77149
**mmu-miR-23b**	5.33056	5.33628	6.29444	6.04286	5.67186
**mmu-miR-132**	3.91552	3.26604	5.24298	3.82069	4.69488
**mmu-miR-29b**	9.19103	9.03144	9.78467	9.69893	9.71282
**mmu-let-7a**	8.67398	8.3786	8.81256	8.88286	8.87949
**mmu-miR-374**	4.86641	5.52888	5.76527	5.23534	4.44956
**mmu-miR-103**	4.96301	5.26604	5.59096	5.90785	5.36702
**mmu-miR-376a**	3.91552	4.75114	4.81916	4.75916	4.21801
**mmu-miR-1902**	2.81558	4.26604	4.854	5.09339	3.86493
**mmu-miR-23a**	3.91552	5.89821	5.70957	6.14221	5.824
**mmu-miR-107**	3.00899	4.5292	4.31976	4.69488	4.39506
**mmu-miR-22**	5.25663	7.03144	6.7654	6.97682	6.44956
**mmu-miR-146a**	5.65249	10.3341	10.2552	10.0085	10.2054
**mmu-miR-21**	7.7021	11.9705	12.2323	11.4491	11.6313
**mmu-miR-27a**	5.40088	6.87467	7.06156	6.90785	6.76075
**mmu-miR-15a**	7.27547	8.75131	8.55175	8.30688	8.17438
**mmu-miR-148a**	4.25626	7.21072	7.58571	5.75889	5.73904
**mmu-miR-106a+17**	7.42652	7.96636	7.89627	7.65328	7.70023
**mmu-miR-301a**	4.00899	4.851	4.98504	4.62761	4.33842
**mmu-miR-96**	2.81558	5.80193	5.31976	4.40531	3.69488
**mmu-miR-24**	3.59335	4.94392	5.36842	4.62761	4.86443

**mmu-let-7i**	5.05311	5.89821	6.30706	5.62761	5.62527
**mmu-miR-484**	4.17871	4.75114	4.95327	4.69488	4.6017
**mmu-let-7d**	7.27547	7.6164	7.43888	7.63626	7.63713
**mmu-miR-350**	3.59335	3.94392	3.854	4.55704	4.6017

*Log2 transformation of Nanostring counts for the 86 miRNAs which passed the minimum intensity filter and for which there were >2 fold differences between any of the samples. The order of rows corresponds to the clustering depicted in Figure 3. B6 naïve T cells are C57BL/6 $CD4^{+}$ T cells that are also $CD44^{lo}CD62L^{hi}$ and B6 memory T cells are C57BL/6 $CD4^{+}$ T cells that are also $CD44^{hi}CD62L^{lo}$. LAT Y136F indicates LAT Y136F $CD4^{+}$ T cells, B6 HP indicates C57BL/6 $CD4^{+}$ T cells undergoing homeostatic proliferation and B6 H poly indicates C57BL/6 $CD4^{+}$ T cells from *H. polygyrus*-infected mice.
