# Supplementary material for: miRNA Signature of Mouse Helper T Cell Hyper-Proliferation
Source: PLoS One. 2013 Jun 25;8(6):e66709. doi: 10.1371/journal.pone.0066709 (PMC3692518; doi:10.1371/journal.pone.0066709)
Supplement: Table S6 — Targets of miRNAs regulated in CD4+ T cells (targets of miRNAs shown in italics may not be direct). Pak1 and Pak2 kinases are equally involved in MAPK transduction and cytoskeletal rearrangement. Pak1 and Pak2, and their upstream regulators such as Vav2, Tiam1, Rac1, Cdc42, and RhoGDI are described in the MAPK table. (PDF) [file pone.0066709.s011.pdf]

Table S6. Targets of miRNAs regulated in $CD4^+$ T cells (targets of miRNAs shown in italics may not be direct)

MAPK TABLE

TARGETS	miRNAs [Refs]	DESCRIPTIONS
CTLA-4	155 [1]	CTLA-4 (Cytotoxic T Lymphocyte Antigen-4) aka CD152 (Cluster of Differentiation 152) is a plasma membrane receptor expressed in T cells. CTLA-4 inhibits the immune system.
TCRα	181a [2]	The TCR (T cell antigen receptor) is a plasma membrane receptor found in T lymphocytes. It recognizes antigenic peptides bound to major histocompatibility complex (MHC) molecules. The TCR is made of two chains. In 95% of T cells, the TCR comprises an α and a β chain, whereas in 5% of T cells, it consists of γ and δ chains. The TCR chains, the ζ-chains, and CD3 (Cluster of Differentiation 3) compose the TCR complex.
CD69	181a [2]	CD69 (Cluster of Differentiation 69) is a transmembrane C-Type lectin receptor. TCR engagement induces early expression of CD69. It is often used as a marker of activation.
ZAP-70	*15a, 16 [3]*	The Syk family Tyr (tyrosine) kinase ZAP-70 (Zeta chain-associated protein kinase 70) plays a critical role in T cell activation. After TCR stimulation, ZAP-70 is phosphorylated on several Tyr residues through autophosphorylation and transphosphorylation by the Src family Tyr kinase Lck. Then the tandem SH2 domains of ZAP-70 engage the doubly phosphorylated ITAMs (Immunoreceptor Tyrosine-based Activation Motif) of TCRζ (hence the abbreviation), positioning ZAP-70 to phosphorylate the adapter LAT (Linker for activation of T cells). In this case, miRNAs-15, -16 increase ZAP-70 levels.
SERCA2a	*22 [4]*	SERCAs (Sarcoplasmic and Endoplasmic Reticulum Ca^{2+} ATPases) are highly conserved Ca^{2+} pumps. SERCA 1 and 2 pumps transport Ca^{2+} from the cytosol to the endoplasmic reticulum lumen against a large concentration gradient. Serca2a activity is decreased in miR-22 KO mice.
Grb2	378 [5]	Grb2 (Growth factor receptor-binding protein 2) is a critical adaptor involved in TCR activation. Grb2 SH2 domain binds to Tyr-phosphorylated LAT and its SH3 domain interacts with Sos. It activates Ras, leading to the activation of the MAPK and PI3K pathways.
PKCε	107 [6]	PKCs (Protein Kinase C) regulate various cellular responses, such as proliferation, apoptosis, and gene expression. PKCs are divided into three groups based on Ca^{2+} dependence and activation mode. Classical PKCs are Ca^{2+}-dependent thanks to their C2 domains and are activated by phosphatidylserine (PS), and diacylglycerol (DAG) or PMA *via* their C1 domains. Novel and atypical PKCs are both Ca^{2+}-independent, but only novel PKCs are activated by PS, DAG, and phorbol esters. PKCε is a novel PKC.

RasGRP1	21 [7]	The RasGRP (Ras Guanyl nucleotide-Releasing Protein) family of GEFs (Guanine nucleotide Exchange Factors) catalyzes the exchange of GDP for GTP on Ras GTPases, promoting their active GTP-bound form. DAG or PMA binding to RasGRP isoforms induces their translocation to the plasma membrane and increases their activity.
p120RasGAP	132 [8]	p120RasGAP (p120 Ras GTPase Activating Protein) aka RASA1 is a 120 kDa cytosolic protein with two main functions: (1) Ras inactivation by increasing its endogenous GTPase activity, through its C-terminal GAP domain and (2) scaffolding function *via* its N-terminal SH2-SH3-SH2 domains.
K-Ras	Let-7 [9], 30c [10], 96 [11]	The 21 kDa small GTPase Ras (K-Ras, H-Ras, and N-Ras) cycle between active (GTP-bound) and inactive (GDP-bound) forms. TCR engagement activates Ras GTPases *via* two GEFs, Sos and RasGRP. Activated Ras stimulates the MAPK cascade and the PI3K pathway.
N-Ras	Let-7 [9], 98 [12]	See K-Ras description above.
B-Raf	*145 [13]*	A-Raf, B-Raf, and c-Raf (Raf-1) are the main effectors recruited by active Ras to stimulate the Erk pathway. c-Raf activation involves phosphorylation at multiple activating sites including Ser 338 (catalytic loop) and Tyr 341. While A-Raf, B-Raf, and c-Raf are similar in sequence and function, they are differentially regulated. Interestingly, B-Raf contains three Akt phosphorylation sites (Ser 364, Ser 428, and Thr 439) and lacks the residue corresponding to Tyr 341 in c-Raf.
Sprouty 2	21 [14]	Sprouty proteins (Spry 1-4) share a highly conserved C-terminal cysteine-rich Spry domain that is critical for their inhibitory function. Single Spry proteins inhibit the Erk pathway at the Raf level, however Spry heterodimers or homodimers have greater inhibitory capacity.
Pak1	7 [15], 98 [12]	The Pak (p21-activated kinase) family of Ser/Thr kinases (Pak1, 2, 3, 4, 5, and 6) is engaged in multiple cellular processes, including cytoskeletal reorganization, MAPK signaling, apoptotic signaling, and growth factor-induced neurite outgrowth. Paks are activated by binding to activated Rac/Cdc42. In addition, Pak1, as opposed to Pak2, is also activated when bound to the SH3 domain of PLC-γ1 [16]; this interaction is favored by the adaptor Bam32. Let-7 is directly repressed by miR-107.
Pak2	23b [17]	Pak2 is cleaved during apoptosis by Caspase 3. The Pak2 C-terminal fragment containing its kinase domain has pro-apoptotic properties.
Rac1	142-3p [18]	Rac and Cdc42 belong to the Rho-GTPase family. Rac1-3 highly similar. Rac1 and Cdc42, the most well characterized members of this group, are ubiquitously expressed. They play key signaling roles in cytoskeletal reorganization, MAPK activation, transcription, and cell growth. GTP binding stimulates the activity of Rac/Cdc42, and the hydrolysis of GTP to GDP thanks to their intrinsic GTPase activity inactive them. GTP hydrolysis is achieved by GAPs, while exchange of GDP for GTP is aided by GEFs.
Cdc42	29 a, b, and c [19]	See Rac1.
RhoGDIA	151 [20]	Rac/Cdc42 activity is also controlled by the binding of RhoGDI, a

		guanine nucleotide dissociation inhibitor, which keeps Rho family GTPases, including Rac and Cdc42, in their inactive GDP-bound state.
Vav2	148a [21]	Vav2 belongs to the Vav oncogene family. Vavs are GEFs for Rac/Cdc42 small GTPases. Unlike Vav1, which is found exclusively in hematopoietic cells, Vav2 is present in most tissues. Vav2 interacts with CD19 and Grb2.
Tiam1	10b [22], *22, 31, 183 [23]*	Tiam family members are GEFs for Rho GTPases. They play an essential role in cytoskeletal rearrangement. In fibroblasts, Tiam1 induces a phenotype similar to that of constitutively activated Rac1. T lymphoma cells overexpressing Tiam1 are invasive, suggesting that the Tiam1-Rac cascade could be involved in the invasion and metastasis of tumors.
Jnk2	17, 106b [24]	Jnk2 (Jun-amino-terminal kinase 2) aka MAPK9 (Mitogen-Activated Protein Kinase 9) is a stress-activated protein kinase. It is involved in apoptosis by phosphorylating pro-apoptotic Bim and FOXOs. It can translocate to the nucleus where it regulates transcription through its phosphorylation on c-Jun, ATF-2, and other transcription factors.
c-Jun	*15a, 16-1 [25]*	c-Jun, JunB, and JunD belong to the Jun family. They are components of the transcription factor AP-1. AP-1 is composed of dimers of c-Fos, c-Jun, JDP and ATF family members. Jnk phosphorylation regulates the transcriptional activity of c-Jun.
HIPK3	106a [26]	HIPK3 (Homeodomain-interacting protein kinase 3) aka Fas-interacting Ser/Thr kinase is a Ser/Thr kinase that regulates transcription and apoptosis. It phosphorylates and activates c-Jun and RUNX2. HIPK3 inhibits apoptosis by phosphorylating FADD.
MKK3/MEK3	*15a, 16-1 [27]*	MKK3 (MAP kinase kinase 3) and MKK6 are two closely related dual-specificity kinases that activate p38 MAP kinase.
MSK1	148a [28]	MSK1 (Mitogen and Stress activated protein Kinase 1) is activated by Erk and p38 in response to TCR engagement, growth factors and cellular stress. MSK1 activates the transcription factor CREB.
IL-2	*146a [29]*	IL-2 (Interleukin-2) is a cytokine that induces T cell proliferation.
PHLPP2	17-5p, 92 [30]	PHLPP1 and PHLPP2 (PH domain and Leucine rich repeat Protein Phosphatases) are phosphatases. They dephosphorylate Akt Ser 473 (hydrophobic motif), thus inactivating the kinase. PHLPPs also dephosphorylate conventional and novel PKCs (hydrophobic motifs).
SHP2	181a [31], 489 [32]	SHP2 aka PTPN11 (Tyr protein phosphatase non-receptor type 11) is a Tyr phosphatase that regulates various cellular processes such as cell growth, differentiation, mitosis, and oncogenic transformation. Gain-of-function mutations in SHP2 cause Noonan and LEOPARD syndromes, because of an abnormal MAPK regulation.
DUSP 1	*96 [33]*	DUSPs (DUal Specificity protein Phosphatases) inactivate their target kinases by dephosphorylating both phosphotyrosines and phosphoserines/threonines. They inhibit MAPK superfamily members (Erk, Jnk, and p38). DUSP family members have different substrate specificities for various MAPKs, various cellular distributions and subcellular localization. DUSP1 aka MKP1 (MAP Kinase Phosphatase 1) is mainly localized in the nucleus and has broad substrate specificity towards Erk, p38, and Jnk.

DUSP 5	181a [31]	DUSP 5 inactivates Erk1.
DUSP 6	181a [31]	DUSP 6 inactivates Erk2.

APOPTOSIS TABLE

TARGETS	miRNAs [Refs}	DESCRIPTIONS
DR4	25 [34]	The cytokine TRAIL is produced and secreted by most normal cells. It induces apoptosis of tumor cells by binding to death receptors. TRAIL presents homology with other members of the tumor necrosis factor (TNF) superfamily. TRAIL binds to the death receptors DR4 (TRAIL-RI) and DR5 (TRAIL-RII). It induces Caspase 8-dependent apoptosis.
Fas	Let-7, 98 [35], 23a [36]	Association of the receptor Fas with its ligand FasL induces apoptosis that plays a critical role in the immune system. Loss of function mutations in either Fas (lpr mice) or FasL (gld mice) lead to lymphadenopathy and splenomegaly due to decreased apoptosis in $CD4^+$ and $CD8^+$ T cells.
FasL	*18a, 19a [37]*	See Fas description above.
FADD	27a [38], 146a [29], 155 [39]	The adaptor FADD (Fas-associated death domain) aka Mort 1 couples apoptotic signals from membrane receptors (Fas, DR4, and DR5 etc.) to Caspase 8.
HIPK3		See description above (MAPK table). In addition to c-Jun phosphorylation, it inhibits apoptosis by phosphorylating FADD.
Caspase 3	Let-7a [40]	Caspases (Cysteine-Aspartic Proteases or Cysteine-dependent Aspartate-directed proteases) are cysteine proteases that play essential roles in apoptosis, necrosis, and inflammation. Caspase 3 is a critical executioner caspase, as it is cleaves many key proteins, such as PARP, the nuclear enzyme poly (ADP-ribose) polymerase.
Caspase 7	23a [41], 106b [42]	Caspase 7 belongs to the caspase family. It also an executioner protease. Like Caspases 2 and 3, Caspase 7 preferentially cleaves substrates following the recognition sequence DEVD.
Caspase 9	24a [43]	Apaf-1 (Apoptotic protease activating factor-1) is a critical signaling protein involved in Caspase 9 activation. Cytosolic Apaf-1 forms a complex with Caspase 9 in the presence of cytochrome c and dATP, ultimately leading to Caspase 9 activation and subsequent activation of Caspase 3. Initiator caspases (Caspases 2, 8, 9, and 10) cleave inactive pro-forms of effector caspases, such as Caspase 3.
Apaf-1	24a [43]	See Caspase 9 above.
Bcl2	7 [44], 15, 16 [45], *21 [46],* 181a [2], 466h [47]	Bcl-2 (B cell lymphoma 2) has a pro-survival function in response to a wide range of apoptotic stimuli by inhibiting the release of cytochrome c.

Bcl-XL	Let-7c and Let-7g [48]	Bcl-XL prevents apoptosis by two different mechanisms: (1) dimerization with a pro-apoptotic molecule inhibits its apoptotic function and (2) decreasing the permeability of mitochondrial outer membrane pores helps maintain a normal membrane state under stressful conditions. Jnk phosphorylates Bcl-XL after drug treatment that destabilizes the cytoskeleton (for example paclitaxel, vinblastine, and nocodazole).
Mcl1	Let-7c and Let-7g [48], *15a, 16-1 [25]*, 29b [49], *101 [50]*, 181b [51], 876-3p [52]	Mcl-1 is an anti-apoptotic member of the Bcl-2 family. Similar to other Bcl-2 family members, Mcl-1 localizes to the mitochondria, interacts with and antagonizes pro-apoptotic Bcl-2 family members and thus inhibits apoptosis induced by cellular stress. Let-7c and Let-7g increase Mcl-1 levels in HepG2 cells.
Blid	*489 [32]*	Blid is a proapoptotic BH3-only member of the Bcl-2 family that interacts with Bcl-XL. Blid overexpression activates Bax and thus increases cytosolic cytochrome c.
BID	*20a [53]*	Bid is a proapoptotic BH3-only member of the Bcl-2 family that interacts with proapoptotic Bax. Cytosolic Bid exists as an inactive precursor and is cleaved by Caspase 8 during apoptosis. Then the C-terminal fragment (tBid) translocates to the mitochondrial outer membrane, inducing the release of cytochrome c.
Bak1	125b [54]	Bak is a proapoptotic member of the Bcl-2 family. During apoptosis, tBID induces conformational changes in Bak to form oligomer channels in the mitochondrial membrane inducing cytochrome c release. Cytosol cytochrome c activates Caspase 9.
Bax	*96 [33]*	During apoptosis, Bax forms oligomers and translocates from the cytosol to the mitochondrial membrane. Bax interaction with pore proteins on the mitochondrial membrane increases membrane permeability, ultimately leading to Caspase 9 activation. Bax and Bak can heterodimerize.
Bim	19 [55], 24 [56], 25 [57], 106b [58], 301a [59]	Bim aka Bcl-2-like 11 or Bam, is a pro-apoptotic BH3-only molecule belonging to Bcl-2 family. Family members including Bad, Bid, or Noxa etc. contain a BH3 domain but lack BH1 or BH2 domains. Bim induces apoptosis by binding to and antagonizing pro-survival molecules of the Bcl-2 family. Bim regulates apoptosis associated with thymocyte negative selection.
Puma	301 [60]	Puma (p53 Upregulated Modulator of Apoptosis) aka BBC3 is a pro-apoptotic BH3-only Bcl-2 family member identified as a p53-inducible gene. Puma α and β bind Bcl-2 and Bcl-XL and promote apoptosis at the mitochondria where Bax and Bak induce release of cytochrome c. Combined loss of Bim and Puma increases numbers of autoreactive thymocytes that escape deletion. Mice deficient for both Puma and Bim spontaneously develop autoimmunity in multiple organs and their T cells transfer organ-specific autoimmunity.
p53	30 a,b,d	The p53 transcription factor is a tumor suppressor that plays a

	[61]	major role in cellular response to DNA damage. p53 activation leads to either cell cycle arrest and DNA repair or apoptosis. MDM2 inhibits p53 accumulation by targeting it for ubiquitination followed by proteasomal degradation. Acetylation of p53 is mediated by p300 and CBP acetyltransferases. Deacetylation of p53 occurs *via* SirT1. The p53 target Puma cooperates with Bim to induce apoptosis at the mitochondria.
MDM2	145 [62]	MDM2 (Mouse Double Minute 2 homolog) aka E3 ubiquitin protein ligase targets p53 to the proteosome. MDM2 functions both as an E3 ubiquitin ligase that interacts with the N-terminal transactivation domain (TAD) of p53 and as an inhibitor of p53 transcription.
MDM4	191 [63]	An additional MDM2 family member, MDM4 aka MDMX, has been discovered and also targets p53 for degradation.
FOXOs		See FOXO description in PI3K/Akt table. FOXOs cooperate with p53 to promote Puma transcription. FOXOs also allow the transcription of FasL, Bim, and BNIP3.
p300		See p300 description in PI3K/Akt table. Among other molecules, p300 regulates p53 and FOXOs.
CDKN1A	17, 19a, 20a [64], 106b [65]	CDKN1A (cyclin-dependent kinase inhibitor 1A) aka p21, is a p53-inducible regulator of cell cycle and cellular survival. Akt phosphorylation of CDKN1A induces its stabilization and promotes survival. Downstream of mTOR, HIF downregulates miRs-17 and -20 which directly target CDKN1A.
BAG3	345 [66]	Decrease in BAG3/4 (Bcl-2 Associated Athanogene 3/4) levels enhance apoptosis mediated by various inducers, while overexpression protects cells from apoptosis. BAG3 sequesters pro-apoptotic Bax in the cytosol, preventing its mitochondrial translocation and its subsequent activation.
BAG4	26a [67]	See BAG3 description above.

PI3K/Akt TABLE

TARGETS	miRNAs [Refs]	DESCRIPTIONS
Ras		See description above (MAPK table). Ras not only activates Raf in the MAPK pathway, but it also activates PI3K.
p85α	29 a, b, and c [19], 376a [68]	Phosphoinositide 3-kinase (PI3K) catalyzes the production of phosphatidylinositol-3,4,5-triphosphate by phosphorylating phosphatidylinositol (PI), phosphatidylinositol-4-phosphate (PIP), and phosphatidylinositol-4,5-bisphosphate (PIP2). PI3Ks comprise a catalytic subunit (p110) and a regulatory subunit (p85).
p110δ	*7 [69]*	PIK3CD gene encodes PI3K catalytic subunit p110δ. In contrast to p110α, and β, p110δ is mainly expressed in leukocytes.
Fli-1	*20a [53],* 145 [70]	Fli-1 (Friend leukemia integration 1 transcription factor) aka ERGB belongs to the Ets family of transcription factors. Fli-1 was first identified in erythroleukemias induced by Friend Murine Leukemia Virus, hence the acronym. Fli-1 inhibits SHIP-1 (Phosphatidylinositol-3,4,5-trisphosphate 5-

		phosphatase 1) expression. SHIP-1 counteracts PI3K activity and thus inhibits Akt activation.
PTEN	17, 106b [24], 18a, 19a [37], 21 [71], 22 [72], 26a [73], 29c [74], 92 [30], 93 [75], 148a [76], 301 [60], 374a [77]	PTEN phosphatase (phosphatase and tensin homologue deleted on chromosome ten) is a tumor suppressor implicated in a wide variety of human cancers. The main substrates of PTEN are inositol phospholipids generated by the PI3K. Thus PTEN is a major negative regulator of the PI3K/Akt pathway. In addition, PTEN regulates p53 activity and levels.
Akt3	93 [78]	Akt isoforms aka PKBs (Protein Kinase B) play a critical role in cell survival. PI3K phosphorylates PIP_2 to generate PIP_3. Once at the plasma membrane *via* binding of PIP_3 to its PH domain, Akt can be phosphorylated by its activating kinases. Critical phosphorylations occur at Thr 308 by PDK1 (activation loop) and at Ser 473 by mTOR (C-ter). Akt inhibits apoptosis by phosphorylation and inactivation of several targets, including Bad, FOXOs, and Caspase 9. In addition, Akt also inhibits mTOR and GSK3 kinases. PTEN and SHIP-1 phosphatases are major inhibitors of the PI3K/Akt cascade During thymocyte development, Akt3 can regulate the double negative to double positive transition. Also Akt3 deficiency worsens experimental autoimmune encephalomyelitis (EAE).
PHLPP2		See description above (MAPK table). PHLPP2 dephosphorylates PKCs (downstream of RasGRP and the CBM complex, which are involved in the MAPK pathway and the NF-κB pathway, respectively). In the PI3K pathway, PHLPP2 dephosphorylates Akt isoforms in their hydrophobic motifs.
FOXO1	27a, 96 [79], 135b [80], 139 [81]	The Forkhead family of transcription factors (FOXO1, FOXO4, and FOXO3a) acts as tumor suppressors by promoting cell cycle arrest and apoptosis. When FOXOs are inactivated by Akt phosphorylation, they are exported from the nucleus, leading to the inhibition of their activity. FOXOs can also be inhibited by the deacetylase sirtuin (SirT1).
FOXO3a	96 [82], 132 [83], 155 [84]	See FOXO1 above.
p300	132 [85]	CBP (CREB-binding protein) and p300 are highly conserved and functionally related transcriptional co-activators. CBP/p300 have histone acetyltransferase (HAT) activity, allowing them to acetylate histones and other proteins. p300 is regulated by PKCs, Akt, and AMPK. Among others, p300 regulates p53, FOXOs, and Rel A (NF-κB).
SirT1	22 [86], 132 [87], 138, 181a [88]	SirT1 is a nuclear enzyme that deacetylates proteins. SirT1 regulates various cellular functions, such as apoptosis, senescence, endocrine signaling, glucose homeostasis, and aging. Targets of SirT1 include p53, p300, FOXOs, and PPARγ. Deacetylation of p53 and FOXOs prevents apoptosis. SirT1 represses the activity of PPARγ in dendritic cells, thereby inducing Th2 skewing.
GSK3β	26a [89]	GSK3 (Glycogen Synthase Kinase-3) is a ubiquitously

		expressed Ser/Thr kinase originally identified as a regulator of glycogen synthesis in response to insulin. GSK-3α and GSK-3β have since been identified as kinases for over forty different targets involved in various pathways, including pro-inflammatory cytokine and interleukin production. GSK3 is inactivated by Akt.
mTOR	*7 [69],* 101 [90]	The mammalian target of rapamycin (mTOR, FRAP, RAFT) is a Ser/Thr kinase that functions as an ATP and amino acid sensor to regulate cellular growth. mTOR autophosphorylates and is phosphorylated by Akt, which activates mTOR to signal to p70S6K.
AMPKα1	19 [55], 148a and not 148b [91]	AMPK (AMP-activated protein Kinase) is a heterotrimeric kinase made of a catalytic α subunit and regulatory β and γ subunits. It has an essential role in energy homeostasis. AMPK is activated by an elevated AMP/ATP ratio. Among all its functions, AMPK regulates protein synthesis and cell growth *via* EF2 and TSC2/mTOR signaling pathways.
p70S6K	*7 [69],* 145 [92]	p70S6 Kinase (p70S6K) is a mitogen activated Ser/Thr kinase that plays a critical role in cell growth and G1 cell cycle progression. p70S6K phosphorylates the S6 protein of the 40S ribosomal subunit.
HIF1α	22 [93], *361 [94]*	HIF1 (Hypoxia-Inducible Factor 1) is a heterodimeric transcription factor that has a critical role in hypoxia. The HIF1 complex contains two subunits, HIF1α and HIF1β. HIF1α regulates the transcription of many genes involved in the responses to hypoxia. HIF1α is rapidly degraded in normoxic cells by the proteosome. HIF1α can also be induced in an oxygen-independent fashion by various cytokines through the PI3K-Akt-mTOR cascade. Utilization of HIF transgenic mice indicates that HIF negatively regulates T cells. HIF1α downregulates miRs-17 and -20a.
HIF1β	107 [95]	See HIF1α above.

NF-κB TABLE

TARGETS	miRNAs [Refs]	DESCRIPTIONS
IKKα	23b [96]	The NF-κB transcription factor is found in an inactive form in the cytosol, bound with the inhibitory IκB proteins. All NF-κB activators induce phosphorylation then proteosomal degradation of IκB. IκB kinase (IKK) complex is made of three IKK subunits and is responsible for IκB phosphorylation. IKKα and IKKβ are the catalytic subunits of the complex and IKKγ (NEMO) is the regulatory subunit.
IKKε	155 [39]	IKKε is essential for the activation NF-κB in the innate immune response. IKKε and TANK-binding kinase 1 activate Akt by direct phosphorylation.
CYLD	19 a and b [99], 181 b1 [98]	CYLD is a cytoplasmic protein containing three cytoskeletal-associated protein glycine conserved (CAP-GLY) domains with deubiquitinating function. Mutations in CYLD are associated with

		cylindromatosis (hence the abbreviation), multiple familial trichoepithelioma, and Brooke-Spiegler syndrome. CYLD regulates inflammation and cell proliferation by removal of ubiquitin chains from several NF-κB pathway proteins. CYLD mediates TCR-induced NF-κB activation *via* the CARMA1/Bcl10/MALT1 (CBM) complex.
A20	19 [99], 125 a and b [100]	A20 aka TNFAIP3 (TNF-α-induced protein 3) activates NF-κB and inhibits apoptosis in response to cytokines. In the NF-κB pathway, A20 Interacts with TRAFs (TNF Receptor Associated Factor) and the NF-κB inhibiting protein ABIN. A20 contains ubiquitin-modifying activity. The N-ter of A20 contains deubiquitinating (DUB) activity for Lys63 branches, such as those found in TRAF6 and RIP, while the C-ter contains ubiquitin ligase (E3) activity for Lys48 branches that leads to proteosomal degradation of substrates.
RIPK1	155 [39]	Ser/Thr kinases RIPKs (Receptor-Interacting Protein) are critical regulators of cellular stress that trigger either pro-survival or pro-apoptotic signals. In addition to the kinase domain, the RIPK death domain interacts with Fas and TNF-R1 *via* TRADD. RIPKs recruit IKKs to the TNF-R1 complex *via* interaction with IKKγ, leading to IκB phosphorylation and degradation. Caspase 8-dependent cleavage of the RIP death domain triggers the apoptotic function of RIP.
TRAF5	*96 [33]*	TRAFs are cytoplasmic adaptors that mediate apoptotic signals from many TNFRs and IL-1R. Recent studies identified six TRAFs (TRAF1-6). TRAF5 is implicated in NF-κB and Jnk activation by TNFRs.
TAB2/3	23b [96]	TAK1 is a MAP3K activated by TGF-β and pro-inflammatory signals. TAK1 is activated after its association with TAK1 binding protein 1 (TAB1). The TAB2 adaptor links TAK1 with TRAF6 and induces TAK1 activation after IL-1 stimulation. Once activated, TAK1 phosphorylates MEK4 and MEK3/6, activating Jnk and p38, respectively. TAK1 and TRAF6 also stimulate the NF-κB cascade by phosphorylating NIK (NF-κB inducing kinase) that activates IKK. TAB3 overexpression activates both NF-κB and AP-1.
NKRF	301a [101]	NKRF (NF-κB-repressing factor) is a transcription factor that interacts with specific negative regulatory elements (NREs). It represses certain NF-κB-dependent genes. NKRF is mainly localized in the nucleolus.
PP2Aε	19 [55]	PP2A (Protein phosphatase type 2A) is an essential Ser/Thr phosphatase conserved in all eukaryotes. PP2A is a key enzyme that regulates fundamental cellular activities such as DNA replication, transcription, translation, metabolism, cell cycle progression, cell division, apoptosis and development. It dephosphorylates CARMA and Akt.

Deacetylase SirT1 is described in the PI3K/Akt table. SirT1 physically interacts with the RelA/p65 subunit of NF-κB and inhibits transcription by deacetylating RelA/p65 at lysine 310.

TRANSCRIPTION FACTOR TABLE

TARGETS	miRNAs [Refs]	DESCRIPTIONS
EOMES	29b [102]	EOMES is transcription factor critical for memory T cells and $CD8^+$ T cells. EOMES is induced in $CD8^+$ T cells after viral and bacterial infection.
Tbet	29b [102]	T-bet aka TBX21 is crucial for the development and the maintenance of type 1 helper T (Th1) cells. T-bet-deficient mice display impaired Th1 differentiation. T-bet induces IFNγ expression.
RUNX3	301a [103]	RUNX3 aka AML2 is a transcription factor that belongs to the Runx family. RUNX3 is a tumor suppressor that binds various transcription factors, including Smads and β-Catenin/TCF4. RUNX3 is also involved in Caspase 3-mediated apoptosis. RUNX3 is normally located in the nucleus, however, in many cancer cells, RUNX3 is Tyr-phosphorylated and found in the cytoplasm where it contributes to tumor progression. RUNX3 is crucial for T cell differentiation. T-bet and RUNX3 cooperate to activate *Ifng* and silence *Il4* in Th1 lymphocytes.
c-Maf	155 [104]	The transcription factor c-Maf controls the production of IL-4 but not other Th2 cytokines. Among other Th2 cytokines, IL-10 is repressed by miR-142 [105].
IRF-1	*301a-3p [106]*	Interferon gamma (IFNγ) is a dimerized soluble cytokine that is critical for innate and adaptive immunity and for tumor suppression. IFNγ is an important activator of macrophages. IFNγ is mainly produced by (1) natural killer (NK) and natural killer T (NKT) cells (innate immunity) and by (2) $CD4^+$ Th1 lymphocytes and $CD8^+$ cytotoxic T cells. It induces IRF transcription factors. IFNγ is directly repressed by miR-29 a, b, and c [107]. IRFs (Interferon Regulatory Factors) compose a family of transcription factors that function within the Jak/Stat pathway to regulate interferon (IFN) and IFN-inducible gene expression. The IRF family contains nine members. IRF-1 transactivates p53 *via* the recruitment of its cofactor p300.
STAT3	17, 20a [108], 93 [78]	STAT3 is critical for signal transduction from many cytokines. STAT3 phosphorylation at Tyr 705 induces dimerization, nuclear translocation, and DNA binding. Transcriptional activity is regulated by phosphorylation at Ser 727 by Erk or mTOR. STAT3 induces T cell survival and inhibits IL-2 production *via* the upregulation of FOXOs.
CREB1	181b [109], 103 [110]	CREB (cAMP Response Element-Binding protein) binds DNA sequences called cAMP Response Elements (CRE). CREB increases the transcription of IL-2, IL-6, IL-10, and TNFα. CREB directly inhibits NF-κB. CREB stimulates proliferation and induces survival of T and B lymphocytes. It regulates Th1, Th2, and Th17 responses. Finally, CREB augments the generation and maintenance of regulatory T cells.
PPARα	21 [111]	Deletion of PPARα (Peroxisome Proliferator-Activated Receptor a) selectively relieves inhibition of NF-κB and c-Jun in male T lymphocytes, resulting in higher production of IFNγ and TNF and lower production of Th2 cytokines. Upon induction of EAE, male

		but not female PPARα$^{-/-}$ mice develop more pronounced disease.
PPARγ	130 a and b [112]	PPARγ activity is inhibited by MAPK phosphorylation at Ser 84. PPARγ regulates apoptosis in T cells. PPARγ mediates potent anti-inflammatory signals.
c-Myc	24 [113], 145 [114]	Members of the Myc/Max/Mad family play a critical role in proliferation, differentiation and apoptosis. Mutations of Myc are found in many cancers, causing the constitutive expression of Myc. This leads to the deregulated expression of many genes, some of which are involved in cell proliferation, and can result in tumor development. Myc regulates expression of 15% of all genes.
MycN	Let-7e, 101 [115]	MycN is a Myc transcription factor family member. See c-Myc description above.
c-Fos	*101 [50],* 139 [116], 146a [29]	The Fos family of nuclear oncogenes includes c-Fos, FosB, Fos-related antigen 1 (FRA1), and Fos-related antigen 2 (FRA2). The rapid and transient expression of Fos is induced by cytokines, growth factors, neurotransmitters, polypeptide hormones, and stress. Fos proteins dimerize with Jun proteins (c-Jun, JunB, and JunD) to form AP-1 (Activator Protein-1), a transcription factor that binds to TRE/AP-1 elements and activates transcription.

Transcription factors Fli-1, HIF1, and FOXOs are presented in the PI3K/Akt table. Transcription factor p53 is described in the Apoptosis table. NF-κB transcription factor and its regulators are detailed in a separate table.

CYTOSKELETON TABLE

TARGETS	miRNAs [Refs]	DESCRIPTIONS
Pyk2	23b [117]	Tyr kinase Pyk2 (Proline-rich tyrosine kinase 2) is homologous to focal adhesion kinase (FAK). Pyk2 is predominantly expressed in hematopoietic cells and in the central nervous system. Pyk2 plays a critical role in MAPK activation, cell spreading, and migration.
RhoB	21 [118]	Rho family small GTPases, including Rho A, B and C, Rac and Cdc42, act as molecular switches, regulating processes such as cell migration, adhesion, proliferation and differentiation.
RhoC	*10b [119]*	See RhoB above. miR-10b suppresses HOXD10, leading to induction of RhoC.
ROCK1	148a [120]	ROCK (Rho-associated kinase), a family of Ser/Thr kinases, is an important downstream target of Rho GTPase and plays an important role in Rho-mediated signaling. The ROCK1 and ROCK2 isoforms are auto-inhibited. Caspase 3-induced cleavage of ROCK1 and cleavage of ROCK2 by granzyme B activate ROCK and lead to phosphorylation of myosin light chain and inhibition of myosin phosphatase. ROCK regulates cytokinesis, cell motility, cell membrane blebbing, and smooth muscle contraction.
IQGAP	98 [12]	IQGAPs are scaffolding proteins involved in cytoskeletal rearrangement. IQGAPs have multiple domains involved in protein interactions and bind to a number of molecules, such as

		actin, myosin light chain, calmodulin, E-cadherin, and β-catenin. Through their GAP-related domains, they bind Rac1/Cdc42. IQGAP1 is ubiquitously expressed and promotes cell polarization and migration. IQGAP1 plays a role in the invasiveness of some cancers.

Pak1 and Pak2 kinases are equally involved in MAPK transduction and cytoskeletal rearrangement. Pak1 and Pak2, and their upstream regulators such as Vav2, Tiam1, Rac1, Cdc42, and RhoGDI are described in the MAPK table.
