# Supplementary material for: miRNA Signature of Mouse Helper T Cell Hyper-Proliferation
Source: PLoS One. 2013 Jun 25;8(6):e66709. doi: 10.1371/journal.pone.0066709 (PMC3692518; doi:10.1371/journal.pone.0066709)
Supplement: Table S7 — Nanostring analysis counts of microRNAs from C57BL/6 naïve CD4+ T Cells*. *miRNAs include all miRNAs reported from Nanostring Technologies including “dead” miRNAs. (PDF) [file pone.0066709.s012.pdf]

Table S7. Nanostring analysis counts of microRNAs from C57BL/6 naïve $CD4^+$ T cells*

miRNA	B6 naive
mmu-miR-720	15,938.21
mmu-miR-150	6,939.45
mmu-miR-142-3p	6,118.55
mmu-miR-16	1,995.92
mmu-miR-1937a+b	1,465.76
mmu-miR-15b	1,065.36
mmu-let-7g	818.892
mmu-miR-29b	584.492
mmu-miR-29a	565.377
mmu-miR-1937c	534.191
mmu-miR-1944	468.8
mmu-let-7a	408.44
mmu-miR-30b	351.097
mmu-let-7f	219.31
mmu-let-7c	208.244
mmu-miR-21	208.244
mmu-miR-181a	203.214
mmu-miR-106a+17	172.028
mmu-miR-25	155.931
mmu-let-7d	154.925
mmu-miR-15a	154.925
mmu-let-7b	129.775
mmu-miR-342-3p	125.751
mmu-miR-26b	115.691
mmu-miR-106b	104.625
mmu-miR-19a	96.577
mmu-miR-669f	93.559
mmu-miR-30d	87.523
mmu-miR-20a+b	86.517
mmu-miR-151-3p	73.439
mmu-miR-297c	72.433
mmu-miR-29c	70.421
mmu-miR-30e	67.403
mmu-miR-151-5p	65.391
mmu-miR-155	62.373
mmu-miR-19b	62.373
mmu-miR-741	61.367
mmu-miR-467f	59.355

mmu-miR-378	56.337
mmu-miR-146a	50.3
mmu-miR-466a-3p+466b-3-3p	50.3
mmu-miR-145	49.294
mmu-miR-10a	45.27
mmu-miR-191	45.27
mmu-miR-27a	42.252
mmu-miR-140	40.24
mmu-miR-23b	40.24
mmu-miR-22	38.228
mmu-miR-361	38.228
mmu-miR-26a	35.21
mmu-miR-2183	34.204
mmu-miR-30c	34.204
mmu-miR-423-5p	34.204
mmu-let-7i	33.198
mmu-miR-338-5p	33.198
mmu-miR-466g	33.198
mmu-miR-547	33.198
mmu-miR-103	31.186
mmu-miR-883b-3p	31.186
mmu-miR-374	29.174
mmu-miR-30a	28.168
mmu-miR-1949	27.162
mmu-miR-219	26.156
mmu-miR-345-3p	26.156
mmu-miR-467e	26.156
mmu-miR-467h+669d+669l	26.156
mmu-miR-669a	26.156
mmu-miR-101b	25.15
mmu-miR-200b	24.144
mmu-miR-340-5p	24.144
mmu-miR-423-3p	24.144
mmu-miR-544	24.144
mmu-miR-425	23.138
mmu-miR-467b	22.132
mmu-miR-98	22.132
mmu-miR-28	21.126
mmu-miR-148b	20.12
mmu-miR-669i	20.12

mmu-miR-743a	20.12
mmu-miR-148a	19.114
mmu-miR-2146	19.114
mmu-miR-467a	19.114
mmu-miR-139-5p	18.108
mmu-miR-3472	18.108
mmu-miR-484	18.108
mmu-miR-33	17.102
mmu-miR-448	17.102
mmu-miR-466d-3p	17.102
mmu-miR-670	17.102
mmu-miR-101a	16.096
mmu-miR-181c	16.096
mmu-miR-301a	16.096
mmu-miR-344	16.096
mmu-miR-409-5p	16.096
mmu-miR-122	15.09
mmu-miR-132	15.09
mmu-miR-135b	15.09
mmu-miR-1839-3p	15.09
mmu-miR-23a	15.09
mmu-miR-323-5p	15.09
mmu-miR-376a	15.09
mmu-miR-465b-5p	15.09
mmu-miR-489	15.09
mmu-miR-539	15.09
mmu-miR-678	15.09
mmu-miR-872	15.09
mmu-miR-876-3p	15.09
mmu-miR-1193	14.084
mmu-miR-124	14.084
mmu-miR-125a-5p	14.084
mmu-miR-1962	14.084
mmu-miR-3471	14.084
mmu-miR-93	14.084
mmu-let-7e	13.078
mmu-miR-130b	13.078
mmu-miR-136	13.078
mmu-miR-153	13.078
mmu-miR-181b+d	13.078
mmu-miR-1893	13.078

mmu-miR-1940	13.078
mmu-miR-1959	13.078
mmu-miR-2132	13.078
mmu-miR-297a+466f+669b	13.078
mmu-miR-323-3p	13.078
mmu-miR-669j	13.078
mmu-miR-695	13.078
mmu-miR-1906	12.072
mmu-miR-24	12.072
mmu-miR-27b	12.072
mmu-miR-329	12.072
mmu-miR-350	12.072
mmu-miR-383	12.072
mmu-miR-450a-5p	12.072
mmu-miR-669o	12.072
mmu-miR-141	11.066
mmu-miR-1939	11.066
mmu-miR-202-3p	11.066
mmu-miR-202-5p	11.066
mmu-miR-206	11.066
mmu-miR-325	11.066
mmu-miR-365	11.066
mmu-miR-532-5p	11.066
mmu-miR-669h-5p	11.066
mmu-miR-683	11.066
mmu-miR-742	11.066
mmu-miR-761	11.066
mmu-miR-1187	10.06
mmu-miR-1194	10.06
mmu-miR-135a	10.06
mmu-miR-144	10.06
mmu-miR-186	10.06
mmu-miR-188-5p	10.06
mmu-miR-196b	10.06
mmu-miR-201	10.06
mmu-miR-291b-3p	10.06
mmu-miR-338-3p	10.06
mmu-miR-380-5p	10.06
mmu-miR-452	10.06
mmu-miR-485	10.06
mmu-miR-488	10.06

mmu-miR-496	10.06
mmu-miR-652	10.06
mmu-miR-690	10.06
mmu-miR-759	10.06
mmu-miR-881	10.06
mmu-miR-1839-5p	9.054
mmu-miR-1929	9.054
mmu-miR-193b	9.054
mmu-miR-1961	9.054
mmu-miR-1963	9.054
mmu-miR-199a-3p	9.054
mmu-miR-211	9.054
mmu-miR-2141	9.054
mmu-miR-302a	9.054
mmu-miR-322	9.054
mmu-miR-3475	9.054
mmu-miR-369-3p	9.054
mmu-miR-410	9.054
mmu-miR-421	9.054
mmu-miR-465a-3p	9.054
mmu-miR-466a-5p+466e-5p	9.054
mmu-miR-467c	9.054
mmu-miR-483	9.054
mmu-miR-493	9.054
mmu-miR-551b	9.054
mmu-miR-574-3p	9.054
mmu-miR-574-5p	9.054
mmu-miR-7a	9.054
mmu-miR-875-5p	9.054
mmu-miR-107	8.048
mmu-miR-128	8.048
mmu-miR-133a	8.048
mmu-miR-152	8.048
mmu-miR-1895	8.048
mmu-miR-1901	8.048
mmu-miR-194	8.048
mmu-miR-1941-3p	8.048
mmu-miR-1943	8.048
mmu-miR-1950	8.048
mmu-miR-1953	8.048
mmu-miR-1960	8.048

mmu-miR-200c	8.048
mmu-miR-204	8.048
mmu-miR-207	8.048
mmu-miR-2138	8.048
mmu-miR-2139	8.048
mmu-miR-2145	8.048
mmu-miR-216b	8.048
mmu-miR-223	8.048
mmu-miR-290-3p	8.048
mmu-miR-302d	8.048
mmu-miR-3072	8.048
mmu-miR-31	8.048
mmu-miR-326	8.048
mmu-miR-328	8.048
mmu-miR-339-3p	8.048
mmu-miR-339-5p	8.048
mmu-miR-345-5p	8.048
mmu-miR-3474	8.048
mmu-miR-367	8.048
mmu-miR-375	8.048
mmu-miR-380-3p	8.048
mmu-miR-450b-5p	8.048
mmu-miR-455	8.048
mmu-miR-463	8.048
mmu-miR-468	8.048
mmu-miR-470	8.048
mmu-miR-501-5p	8.048
mmu-miR-511	8.048
mmu-miR-669e	8.048
mmu-miR-706	8.048
mmu-miR-744	8.048
mmu-miR-770-3p	8.048
mmu-miR-873	8.048
mmu-miR-883a-3p	8.048
mmu-miR-92a	8.048
mmu-miR-99a	8.048
mmu-miR-125a-3p	7.042
mmu-miR-154	7.042
mmu-miR-190	7.042
mmu-miR-1902	7.042
mmu-miR-1928	7.042

mmu-miR-1936	7.042
mmu-miR-195	7.042
mmu-miR-1957	7.042
mmu-miR-1965	7.042
mmu-miR-203	7.042
mmu-miR-2137	7.042
mmu-miR-2861	7.042
mmu-miR-291a-3p	7.042
mmu-miR-296-5p	7.042
mmu-miR-335-3p	7.042
mmu-miR-337-3p	7.042
mmu-miR-337-5p	7.042
mmu-miR-34b-3p	7.042
mmu-miR-362-3p	7.042
mmu-miR-466d-5p	7.042
mmu-miR-467g	7.042
mmu-miR-495	7.042
mmu-miR-540-3p	7.042
mmu-miR-541	7.042
mmu-miR-664	7.042
mmu-miR-668	7.042
mmu-miR-669g	7.042
mmu-miR-673-5p	7.042
mmu-miR-674	7.042
mmu-miR-677	7.042
mmu-miR-688	7.042
mmu-miR-694	7.042
mmu-miR-710	7.042
mmu-miR-717	7.042
mmu-miR-719	7.042
mmu-miR-762	7.042
mmu-miR-770-5p	7.042
mmu-miR-7b	7.042
mmu-miR-96	7.042
mmu-miR-1198	6.036
mmu-miR-1306	6.036
mmu-miR-146b	6.036
mmu-miR-187	6.036
mmu-miR-1897-3p	6.036
mmu-miR-1898	6.036
mmu-miR-1903	6.036

mmu-miR-192	6.036
mmu-miR-1935	6.036
mmu-miR-1941-5p	6.036
mmu-miR-1942	6.036
mmu-miR-1945	6.036
mmu-miR-1964	6.036
mmu-miR-200a	6.036
mmu-miR-205	6.036
mmu-miR-294	6.036
mmu-miR-297b-3p	6.036
mmu-miR-324-5p	6.036
mmu-miR-331-3p	6.036
mmu-miR-342-5p	6.036
mmu-miR-343	6.036
mmu-miR-3473	6.036
mmu-miR-34b-5p	6.036
mmu-miR-382	6.036
mmu-miR-384-5p	6.036
mmu-miR-412	6.036
mmu-miR-431	6.036
mmu-miR-434-5p	6.036
mmu-miR-449a	6.036
mmu-miR-450b-3p	6.036
mmu-miR-464	6.036
mmu-miR-466j	6.036
mmu-miR-466k	6.036
mmu-miR-499	6.036
mmu-miR-500	6.036
mmu-miR-542-3p	6.036
mmu-miR-653	6.036
mmu-miR-675-5p	6.036
mmu-miR-696	6.036
mmu-miR-708	6.036
mmu-miR-713	6.036
mmu-miR-743b-3p	6.036
mmu-miR-760	6.036
mmu-miR-764-5p	6.036
mmu-miR-802	6.036
mmu-miR-875-3p	6.036
mmu-miR-878-3p	6.036
mmu-miR-879	6.036

mmu-miR-1186	5.03
mmu-miR-1186b	5.03
mmu-miR-1224	5.03
mmu-miR-125b-3p	5.03
mmu-miR-126-3p	5.03
mmu-miR-1274a	5.03
mmu-miR-129-3p	5.03
mmu-miR-129-5p	5.03
mmu-miR-143	5.03
mmu-miR-149	5.03
mmu-miR-184	5.03
mmu-miR-1894-5p	5.03
mmu-miR-1896	5.03
mmu-miR-18a	5.03
mmu-miR-1905	5.03
mmu-miR-1931	5.03
mmu-miR-1946a	5.03
mmu-miR-1966	5.03
mmu-miR-1968	5.03
mmu-miR-1969	5.03
mmu-miR-1982	5.03
mmu-miR-214	5.03
mmu-miR-220	5.03
mmu-miR-293	5.03
mmu-miR-3099	5.03
mmu-miR-331-5p	5.03
mmu-miR-340-3p	5.03
mmu-miR-341	5.03
mmu-miR-346	5.03
mmu-miR-377	5.03
mmu-miR-409-3p	5.03
mmu-miR-432	5.03
mmu-miR-465a-5p	5.03
mmu-miR-466h	5.03
mmu-miR-471	5.03
mmu-miR-486	5.03
mmu-miR-494	5.03
mmu-miR-497	5.03
mmu-miR-540-5p	5.03
mmu-miR-543	5.03
mmu-miR-582-3p	5.03

mmu-miR-590-5p	5.03
mmu-miR-654-3p	5.03
mmu-miR-667	5.03
mmu-miR-704	5.03
mmu-miR-718	5.03
mmu-miR-764-3p	5.03
mmu-miR-804	5.03
mmu-miR-874	5.03
mmu-miR-878-5p	5.03
mmu-miR-882	5.03
mmu-miR-883a-5p	5.03
mmu-miR-883b-5p	5.03
mmu-miR-92b	5.03
mmu-miR-99b	5.03
mmu-miR-1	4.024
mmu-miR-105	4.024
mmu-miR-1197	4.024
mmu-miR-1199	4.024
mmu-miR-125b-5p	4.024
mmu-miR-133b	4.024
mmu-miR-147	4.024
mmu-miR-182	4.024
mmu-miR-183	4.024
mmu-miR-1900	4.024
mmu-miR-1907	4.024
mmu-miR-190b	4.024
mmu-miR-193	4.024
mmu-miR-1933-5p	4.024
mmu-miR-1956	4.024
mmu-miR-1983	4.024
mmu-miR-199a-5p	4.024
mmu-miR-208b	4.024
mmu-miR-2133	4.024
mmu-miR-2135	4.024
mmu-miR-2136	4.024
mmu-miR-221	4.024
mmu-miR-222	4.024
mmu-miR-290-5p	4.024
mmu-miR-291b-5p	4.024
mmu-miR-292-5p	4.024
mmu-miR-295	4.024

mmu-miR-296-3p	4.024
mmu-miR-298	4.024
mmu-miR-300	4.024
mmu-miR-301b	4.024
mmu-miR-320	4.024
mmu-miR-324-3p	4.024
mmu-miR-3470a+b	4.024
mmu-miR-34a	4.024
mmu-miR-351	4.024
mmu-miR-369-5p	4.024
mmu-miR-376b	4.024
mmu-miR-379	4.024
mmu-miR-411	4.024
mmu-miR-449b	4.024
mmu-miR-465c-5p	4.024
mmu-miR-466i	4.024
mmu-miR-467d	4.024
mmu-miR-532-3p	4.024
mmu-miR-542-5p	4.024
mmu-miR-546	4.024
mmu-miR-568	4.024
mmu-miR-582-5p	4.024
mmu-miR-599	4.024
mmu-miR-671-3p	4.024
mmu-miR-676	4.024
mmu-miR-684	4.024
mmu-miR-686	4.024
mmu-miR-689	4.024
mmu-miR-692	4.024
mmu-miR-693-3p	4.024
mmu-miR-698	4.024
mmu-miR-703	4.024
mmu-miR-709	4.024
mmu-miR-767	4.024
mmu-miR-876-5p	4.024
mmu-miR-880	4.024
mmu-miR-10b	3.018
mmu-miR-1188	3.018
mmu-miR-1190	3.018
mmu-miR-1191	3.018
mmu-miR-126-5p	3.018

mmu-miR-127	3.018
mmu-miR-130a	3.018
mmu-miR-134	3.018
mmu-miR-137	3.018
mmu-miR-1892	3.018
mmu-miR-1894-3p	3.018
mmu-miR-1927	3.018
mmu-miR-1934	3.018
mmu-miR-1938	3.018
mmu-miR-1946b	3.018
mmu-miR-1947	3.018
mmu-miR-1951	3.018
mmu-miR-1952	3.018
mmu-miR-1967	3.018
mmu-miR-1970	3.018
mmu-miR-210	3.018
mmu-miR-212	3.018
mmu-miR-216a	3.018
mmu-miR-217	3.018
mmu-miR-292-3p	3.018
mmu-miR-32	3.018
mmu-miR-327	3.018
mmu-miR-330	3.018
mmu-miR-34c	3.018
mmu-miR-381	3.018
mmu-miR-384-3p	3.018
mmu-miR-433	3.018
mmu-miR-434-3p	3.018
mmu-miR-451	3.018
mmu-miR-453	3.018
mmu-miR-466c-5p	3.018
mmu-miR-466l	3.018
mmu-miR-469	3.018
mmu-miR-487b	3.018
mmu-miR-490	3.018
mmu-miR-592	3.018
mmu-miR-654-5p	3.018
mmu-miR-697	3.018
mmu-miR-707	3.018
mmu-miR-711	3.018
mmu-miR-712	3.018

mmu-miR-743b-5p	3.018
mmu-miR-758	3.018
mmu-miR-871	3.018
mmu-miR-877	3.018
mmu-miR-1192	2.012
mmu-miR-1195	2.012
mmu-miR-1196	2.012
mmu-miR-138	2.012
mmu-miR-185	2.012
mmu-miR-188-3p	2.012
mmu-miR-1904	2.012
mmu-miR-1932	2.012
mmu-miR-1933-3p	2.012
mmu-miR-1955	2.012
mmu-miR-1958	2.012
mmu-miR-1971	2.012
mmu-miR-1981	2.012
mmu-miR-2134	2.012
mmu-miR-2140	2.012
mmu-miR-2182	2.012
mmu-miR-224	2.012
mmu-miR-291a-5p	2.012
mmu-miR-297b-5p	2.012
mmu-miR-302c	2.012
mmu-miR-335-5p	2.012
mmu-miR-362-5p	2.012
mmu-miR-363	2.012
mmu-miR-376c	2.012
mmu-miR-429	2.012
mmu-miR-449c	2.012
mmu-miR-466f-5p	2.012
mmu-miR-503	2.012
mmu-miR-505	2.012
mmu-miR-509-5p	2.012
mmu-miR-615-3p	2.012
mmu-miR-615-5p	2.012
mmu-miR-665	2.012
mmu-miR-666-3p	2.012
mmu-miR-666-5p	2.012
mmu-miR-669m	2.012
mmu-miR-671-5p	2.012

mmu-miR-672	2.012
mmu-miR-679	2.012
mmu-miR-693-5p	2.012
mmu-miR-700	2.012
mmu-miR-701	2.012
mmu-miR-702	2.012
mmu-miR-715	2.012
mmu-miR-9	2.012
mmu-miR-100	1.006
mmu-miR-139-3p	1.006
mmu-miR-142-5p	1.006
mmu-miR-1899	1.006
mmu-miR-18b	1.006
mmu-miR-1930	1.006
mmu-miR-1948	1.006
mmu-miR-1954	1.006
mmu-miR-196a	1.006
mmu-miR-208a	1.006
mmu-miR-218	1.006
mmu-miR-299	1.006
mmu-miR-302b	1.006
mmu-miR-370	1.006
mmu-miR-450a-3p	1.006
mmu-miR-491	1.006
mmu-miR-501-3p	1.006
mmu-miR-504	1.006
mmu-miR-509-3p	1.006
mmu-miR-590-3p	1.006
mmu-miR-673-3p	1.006
mmu-miR-675-3p	1.006
mmu-miR-680	1.006
mmu-miR-681	1.006
mmu-miR-682	1.006
mmu-miR-687	1.006
mmu-miR-691	1.006
mmu-miR-714	1.006
mmu-miR-763	1.006

*miRNAs include all miRNAs reported from Nanostring Technologies including “dead” miRNAs.
