# Supplementary material for: miRNA Signature of Mouse Helper T Cell Hyper-Proliferation
Source: PLoS One. 2013 Jun 25;8(6):e66709. doi: 10.1371/journal.pone.0066709 (PMC3692518; doi:10.1371/journal.pone.0066709)
Supplement: References S1 — Supplemental Biography. (PDF) [file pone.0066709.s013.pdf]

SUPPLEMENTAL BIBLIOGRAPHY

1. Sonkoly E, Janson P, Majuri ML, Savinko T, Fyhrquist N, et al. (2010) MiR-155 is overexpressed in patients with atopic dermatitis and modulates T-cell proliferative responses by targeting cytotoxic T lymphocyte-associated antigen 4. J Allergy Clin Immunol 126: 581-589 e581-520.
2. Neilson JR, Zheng GX, Burge CB, Sharp PA (2007) Dynamic regulation of miRNA expression in ordered stages of cellular development. Genes Dev 21: 578-589.
3. Fabbri M, Bottoni A, Shimizu M, Spizzo R, Nicoloso MS, et al. (2011) Association of a microRNA/TP53 feedback circuitry with pathogenesis and outcome of B-cell chronic lymphocytic leukemia. JAMA 305: 59-67.
4. Gurha P, Abreu-Goodger C, Wang T, Ramirez MO, Drumond AL, et al. (2012) Targeted deletion of microRNA-22 promotes stress-induced cardiac dilation and contractile dysfunction. Circulation 125: 2751-2761.
5. Nagalingam RS, Sundaresan NR, Gupta MP, Geenen D, Solaro RJ, et al. (2013) A cardiac enriched microRNA, miR-378 blocks cardiac hypertrophy by targeting Ras-signaling. J Biol Chem.
6. Datta J, Smith A, Lang JC, Islam M, Dutt D, et al. (2012) microRNA-107 functions as a candidate tumor-suppressor gene in head and neck squamous cell carcinoma by downregulation of protein kinase Cvarepsilon. Oncogene 31: 4045-4053.
7. Pan W, Zhu S, Yuan M, Cui H, Wang L, et al. (2010) MicroRNA-21 and microRNA-148a contribute to DNA hypomethylation in lupus CD4+ T cells by directly and indirectly targeting DNA methyltransferase 1. J Immunol 184: 6773-6781.
8. Anand S, Majeti BK, Acevedo LM, Murphy EA, Mukthavaram R, et al. (2010) MicroRNA-132-mediated loss of p120RasGAP activates the endothelium to facilitate pathological angiogenesis. Nat Med 16: 909-914.
9. Johnson SM, Grosshans H, Shingara J, Byrom M, Jarvis R, et al. (2005) RAS is regulated by the let-7 microRNA family. Cell 120: 635-647.
10. Tanic M, Yanowsky K, Rodriguez-Antona C, Andres R, Marquez-Rodas I, et al. (2012) Deregulated miRNAs in hereditary breast cancer revealed a role for miR-30c in regulating KRAS oncogene. PLoS One 7: e38847.
11. Yu S, Lu Z, Liu C, Meng Y, Ma Y, et al. (2010) miRNA-96 suppresses KRAS and functions as a tumor suppressor gene in pancreatic cancer. Cancer Res 70: 6015-6025.
12. Gennarino VA, Sardiello M, Avellino R, Meola N, Maselli V, et al. (2009) MicroRNA target prediction by expression analysis of host genes. Genome Res 19: 481-490.
13. Pagliuca A, Valvo C, Fabrizi E, di Martino S, Biffoni M, et al. (2012) Analysis of the combined action of miR-143 and miR-145 on oncogenic pathways in colorectal cancer cells reveals a coordinate program of gene repression. Oncogene.
14. Sayed D, Rane S, Lypowy J, He M, Chen IY, et al. (2008) MicroRNA-21 targets Sprouty2 and promotes cellular outgrowths. Mol Biol Cell 19: 3272-3282.
15. Reddy SD, Ohshiro K, Rayala SK, Kumar R (2008) MicroRNA-7, a homeobox D10 target, inhibits p21-activated kinase 1 and regulates its functions. Cancer Res 68: 8195-8200.
16. Rouquette-Jazdanian AK, Sommers CL, Kortum RL, Morrison DK, Samelson LE (2012) LAT-independent Erk activation via Bam32-PLC-gamma1-Pak1 complexes: GTPase-independent Pak1 activation. Mol Cell 48: 298-312.
17. Zhang H, Hao Y, Yang J, Zhou Y, Li J, et al. (2011) Genome-wide functional screening of miR-23b as a pleiotropic modulator suppressing cancer metastasis. Nat Commun 2: 554.
18. Wu L, Cai C, Wang X, Liu M, Li X, et al. (2011) MicroRNA-142-3p, a new regulator of RAC1, suppresses the migration and invasion of hepatocellular carcinoma cells. FEBS Lett 585: 1322-1330.
19. Park SY, Lee JH, Ha M, Nam JW, Kim VN (2009) miR-29 miRNAs activate p53 by targeting p85 alpha and CDC42. Nat Struct Mol Biol 16: 23-29.
20. Ding J, Huang S, Wu S, Zhao Y, Liang L, et al. (2010) Gain of miR-151 on chromosome 8q24.3 facilitates tumour cell migration and spreading through downregulating RhoGDIA. Nat Cell Biol 12: 390-399.

21. Tseng CW, Lin CC, Chen CN, Huang HC, Juan HF (2011) Integrative network analysis reveals active microRNAs and their functions in gastric cancer. BMC Syst Biol 5: 99.
22. Moriarty CH, Pursell B, Mercurio AM (2010) miR-10b targets Tiam1: implications for Rac activation and carcinoma migration. J Biol Chem 285: 20541-20546.
23. Li J, Liang S, Jin H, Xu C, Ma D, et al. (2012) Tiam1, negatively regulated by miR-22, miR-183 and miR-31, is involved in migration, invasion and viability of ovarian cancer cells. Oncol Rep 27: 1835-1842.
24. Trompeter HI, Abbad H, Iwaniuk KM, Hafner M, Renwick N, et al. (2011) MicroRNAs MiR-17, MiR-20a, and MiR-106b act in concert to modulate E2F activity on cell cycle arrest during neuronal lineage differentiation of USSC. PLoS One 6: e16138.
25. Calin GA, Cimmino A, Fabbri M, Ferracin M, Wojcik SE, et al. (2008) MiR-15a and miR-16-1 cluster functions in human leukemia. Proc Natl Acad Sci U S A 105: 5166-5171.
26. Landais S, Landry S, Legault P, Rassart E (2007) Oncogenic potential of the miR-106-363 cluster and its implication in human T-cell leukemia. Cancer Res 67: 5699-5707.
27. Hanlon K, Rudin CE, Harries LW (2009) Investigating the targets of MIR-15a and MIR-16-1 in patients with chronic lymphocytic leukemia (CLL). PLoS One 4: e7169.
28. Fujita Y, Kojima K, Ohhashi R, Hamada N, Nozawa Y, et al. (2010) MiR-148a attenuates paclitaxel resistance of hormone-refractory, drug-resistant prostate cancer PC3 cells by regulating MSK1 expression. J Biol Chem 285: 19076-19084.
29. Curtale G, Citarella F, Carissimi C, Goldoni M, Carucci N, et al. (2010) An emerging player in the adaptive immune response: microRNA-146a is a modulator of IL-2 expression and activation-induced cell death in T lymphocytes. Blood 115: 265-273.
30. Rao E, Jiang C, Ji M, Huang X, Iqbal J, et al. (2012) The miRNA-17 approximately 92 cluster mediates chemoresistance and enhances tumor growth in mantle cell lymphoma via PI3K/AKT pathway activation. Leukemia 26: 1064-1072.
31. Li QJ, Chau J, Ebert PJ, Sylvester G, Min H, et al. (2007) miR-181a is an intrinsic modulator of T cell sensitivity and selection. Cell 129: 147-161.
32. Kikkawa N, Hanazawa T, Fujimura L, Nohata N, Suzuki H, et al. (2010) miR-489 is a tumour-suppressive miRNA target PTPN11 in hypopharyngeal squamous cell carcinoma (HSCC). Br J Cancer 103: 877-884.
33. Yamada Y, Enokida H, Kojima S, Kawakami K, Chiyomaru T, et al. (2011) MiR-96 and miR-183 detection in urine serve as potential tumor markers of urothelial carcinoma: correlation with stage and grade, and comparison with urinary cytology. Cancer Sci 102: 522-529.
34. Razumilava N, Bronk SF, Smoot RL, Fingas CD, Werneburg NW, et al. (2011) miR-25 targets TNF-related apoptosis inducing ligand (TRAIL) death receptor-4 and promotes apoptosis resistance in cholangiocarcinoma. Hepatology 55: 465-475.
35. Wang S, Tang Y, Cui H, Zhao X, Luo X, et al. (2011) Let-7/miR-98 regulate Fas and Fas-mediated apoptosis. Genes Immun 12: 149-154.
36. Lin H, Qian J, Castillo AC, Long B, Keyes KT, et al. (2011) Effect of miR-23 on oxidant-induced injury in human retinal pigment epithelial cells. Invest Ophthalmol Vis Sci 52: 6308-6314.
37. Liu XS, Chopp M, Wang XL, Zhang L, Hozeska-Solgot A, et al. (2013) MicroRNA-17/92 cluster mediates the proliferation and survival of neural progenitor cells after stroke. J Biol Chem.
38. Chhabra R, Adlakha YK, Hariharan M, Scaria V, Saini N (2009) Upregulation of miR-23a-27a-24-2 cluster induces caspase-dependent and -independent apoptosis in human embryonic kidney cells. PLoS One 4: e5848.
39. Tili E, Michaille JJ, Cimino A, Costinean S, Dumitru CD, et al. (2007) Modulation of miR-155 and miR-125b levels following lipopolysaccharide/TNF-alpha stimulation and their possible roles in regulating the response to endotoxin shock. J Immunol 179: 5082-5089.
40. Tsang WP, Kwok TT (2008) Let-7a microRNA suppresses therapeutics-induced cancer cell death by targeting caspase-3. Apoptosis 13: 1215-1222.
41. Ruan W, Xu JM, Li SB, Yuan LQ, Dai RP (2012) Effects of down-regulation of microRNA-23a on TNF-alpha-induced endothelial cell apoptosis through caspase-dependent pathways. Cardiovasc Res 93: 623-632.

42. Hudson RS, Yi M, Esposito D, Glynn SA, Starks AM, et al. (2012) MicroRNA-106b-25 cluster expression is associated with early disease recurrence and targets caspase-7 and focal adhesion in human prostate cancer. Oncogene.
43. Walker JC, Harland RM (2009) microRNA-24a is required to repress apoptosis in the developing neural retina. Genes Dev 23: 1046-1051.
44. Xiong S, Zheng Y, Jiang P, Liu R, Liu X, et al. (2011) MicroRNA-7 inhibits the growth of human non-small cell lung cancer A549 cells through targeting BCL-2. Int J Biol Sci 7: 805-814.
45. Cimmino A, Calin GA, Fabbri M, Iorio MV, Ferracin M, et al. (2005) miR-15 and miR-16 induce apoptosis by targeting BCL2. Proc Natl Acad Sci U S A 102: 13944-13949.
46. Wickramasinghe NS, Manavalan TT, Dougherty SM, Riggs KA, Li Y, et al. (2009) Estradiol downregulates miR-21 expression and increases miR-21 target gene expression in MCF-7 breast cancer cells. Nucleic Acids Res 37: 2584-2595.
47. Druz A, Chu C, Majors B, Santuary R, Betenbaugh M, et al. (2011) A novel microRNA mmu-miR-466h affects apoptosis regulation in mammalian cells. Biotechnol Bioeng 108: 1651-1661.
48. Shimizu S, Takehara T, Hikita H, Kodama T, Miyagi T, et al. (2010) The let-7 family of microRNAs inhibits Bcl-xL expression and potentiates sorafenib-induced apoptosis in human hepatocellular carcinoma. J Hepatol 52: 698-704.
49. Mott JL, Kobayashi S, Bronk SF, Gores GJ (2007) mir-29 regulates Mcl-1 protein expression and apoptosis. Oncogene 26: 6133-6140.
50. Wang HJ, Ruan HJ, He XJ, Ma YY, Jiang XT, et al. (2010) MicroRNA-101 is down-regulated in gastric cancer and involved in cell migration and invasion. Eur J Cancer.
51. Visone R, Veronese A, Rassenti LZ, Balatti V, Pearl DK, et al. (2011) miR-181b is a biomarker of disease progression in chronic lymphocytic leukemia. Blood 118: 3072-3079.
52. Lam LT, Lu X, Zhang H, Lesniewski R, Rosenberg S, et al. (2010) A microRNA screen to identify modulators of sensitivity to BCL2 inhibitor ABT-263 (navitoclax). Mol Cancer Ther 9: 2943-2950.
53. Cox MB, Cairns MJ, Gandhi KS, Carroll AP, Moscovis S, et al. (2010) MicroRNAs miR-17 and miR-20a inhibit T cell activation genes and are under-expressed in MS whole blood. PLoS One 5: e12132.
54. Zhou M, Liu Z, Zhao Y, Ding Y, Liu H, et al. (2010) MicroRNA-125b confers the resistance of breast cancer cells to paclitaxel through suppression of pro-apoptotic Bcl-2 antagonist killer 1 (Bak1) expression. J Biol Chem 285: 21496-21507.
55. Mavrakis KJ, Wolfe AL, Oricchio E, Palomero T, de Keersmaecker K, et al. (2010) Genome-wide RNA-mediated interference screen identifies miR-19 targets in Notch-induced T-cell acute lymphoblastic leukaemia. Nat Cell Biol 12: 372-379.
56. Qian L, Van Laake LW, Huang Y, Liu S, Wendland MF, et al. (2011) miR-24 inhibits apoptosis and represses Bim in mouse cardiomyocytes. J Exp Med 208: 549-560.
57. Zhang H, Zuo Z, Lu X, Wang L, Wang H, et al. (2012) MiR-25 regulates apoptosis by targeting Bim in human ovarian cancer. Oncol Rep 27: 594-598.
58. Gupta S, Read DE, Deepti A, Cawley K, Gupta A, et al. (2012) Perk-dependent repression of miR-106b-25 cluster is required for ER stress-induced apoptosis. Cell Death Dis 3: e333.
59. Chen Z, Chen LY, Dai HY, Wang P, Gao S, et al. (2012) miR-301a promotes pancreatic cancer cell proliferation by directly inhibiting Bim expression. J Cell Biochem 113: 3229-3235.
60. Shi W, Gerster K, Alajez NM, Tsang J, Waldron L, et al. (2011) MicroRNA-301 mediates proliferation and invasion in human breast cancer. Cancer Res 71: 2926-2937.
61. Li J, Donath S, Li Y, Qin D, Prabhakar BS, et al. (2010) miR-30 regulates mitochondrial fission through targeting p53 and the dynamin-related protein-1 pathway. PLoS Genet 6: e1000795.
62. Zhang J, Sun Q, Zhang Z, Ge S, Han ZG, et al. (2013) Loss of microRNA-143/145 disturbs cellular growth and apoptosis of human epithelial cancers by impairing the MDM2-p53 feedback loop. Oncogene 32: 61-69.

63. Wynendaele J, Bohnke A, Leucci E, Nielsen SJ, Lambertz I, et al. (2010) An illegitimate microRNA target site within the 3' UTR of MDM4 affects ovarian cancer progression and chemosensitivity. Cancer Res 70: 9641-9649.
64. Inomata M, Tagawa H, Guo YM, Kameoka Y, Takahashi N, et al. (2009) MicroRNA-17-92 down-regulates expression of distinct targets in different B-cell lymphoma subtypes. Blood 113: 396-402.
65. Ivanovska I, Ball AS, Diaz RL, Magnus JF, Kibukawa M, et al. (2008) MicroRNAs in the miR-106b family regulate p21/CDKN1A and promote cell cycle progression. Mol Cell Biol 28: 2167-2174.
66. Tang JT, Wang JL, Du W, Hong J, Zhao SL, et al. (2011) MicroRNA 345, a methylation-sensitive microRNA is involved in cell proliferation and invasion in human colorectal cancer. Carcinogenesis 32: 1207-1215.
67. Reuland SN, Smith SM, Bemis LT, Goldstein NB, Almeida AR, et al. (2012) MicroRNA-26a Is Strongly Downregulated in Melanoma and Induces Cell Death through Repression of Silencer of Death Domains (SODD). J Invest Dermatol.
68. Zheng Y, Yin L, Chen H, Yang S, Pan C, et al. (2012) miR-376a suppresses proliferation and induces apoptosis in hepatocellular carcinoma. FEBS Lett 586: 2396-2403.
69. Fang Y, Xue JL, Shen Q, Chen J, Tian L (2012) MicroRNA-7 inhibits tumor growth and metastasis by targeting the phosphoinositide 3-kinase/Akt pathway in hepatocellular carcinoma. Hepatology 55: 1852-1862.
70. Zhang J, Guo H, Zhang H, Wang H, Qian G, et al. (2011) Putative tumor suppressor miR-145 inhibits colon cancer cell growth by targeting oncogene Friend leukemia virus integration 1 gene. Cancer 117: 86-95.
71. Zhang JG, Wang JJ, Zhao F, Liu Q, Jiang K, et al. (2010) MicroRNA-21 (miR-21) represses tumor suppressor PTEN and promotes growth and invasion in non-small cell lung cancer (NSCLC). Clin Chim Acta 411: 846-852.
72. Bar N, Dikstein R (2010) miR-22 forms a regulatory loop in PTEN/AKT pathway and modulates signaling kinetics. PLoS One 5: e10859.
73. Huse JT, Brennan C, Hambardzumyan D, Wee B, Pena J, et al. (2009) The PTEN-regulating microRNA miR-26a is amplified in high-grade glioma and facilitates gliomagenesis in vivo. Genes Dev 23: 1327-1337.
74. Tumaneng K, Schlegelmilch K, Russell RC, Yimlamai D, Basnet H, et al. (2012) YAP mediates crosstalk between the Hippo and PI(3)K-TOR pathways by suppressing PTEN via miR-29. Nat Cell Biol 14: 1322-1329.
75. Fu X, Tian J, Zhang L, Chen Y, Hao Q (2012) Involvement of microRNA-93, a new regulator of PTEN/Akt signaling pathway, in regulation of chemotherapeutic drug cisplatin chemosensitivity in ovarian cancer cells. FEBS Lett 586: 1279-1286.
76. Yuan K, Lian Z, Sun B, Clayton MM, Ng IO, et al. (2012) Role of miR-148a in hepatitis B associated hepatocellular carcinoma. PLoS One 7: e35331.
77. Cai J, Guan H, Fang L, Yang Y, Zhu X, et al. (2013) MicroRNA-374a activates Wnt/beta-catenin signaling to promote breast cancer metastasis. J Clin Invest.
78. Liu S, Patel SH, Ginestier C, Ibarra I, Martin-Trevino R, et al. (2012) MicroRNA93 regulates proliferation and differentiation of normal and malignant breast stem cells. PLoS Genet 8: e1002751.
79. Guttilla IK, White BA (2009) Coordinate regulation of FOXO1 by miR-27a, miR-96, and miR-182 in breast cancer cells. J Biol Chem 284: 23204-23216.
80. Matsuyama H, Suzuki HI, Nishimori H, Noguchi M, Yao T, et al. (2011) miR-135b mediates NPM-ALK-driven oncogenicity and renders IL-17-producing immunophenotype to anaplastic large cell lymphoma. Blood 118: 6881-6892.
81. Hasseine LK, Hinault C, Lebrun P, Gautier N, Paul-Bellon R, et al. (2009) miR-139 impacts FoxO1 action by decreasing FoxO1 protein in mouse hepatocytes. Biochem Biophys Res Commun 390: 1278-1282.
82. Lin H, Dai T, Xiong H, Zhao X, Chen X, et al. (2010) Unregulated miR-96 induces cell proliferation in human breast cancer by downregulating transcriptional factor FOXO3a. PLoS One 5: e15797.

83. Ucar A, Gupta SK, Fiedler J, Erikci E, Kardasinski M, et al. (2012) The miRNA-212/132 family regulates both cardiac hypertrophy and cardiomyocyte autophagy. Nat Commun 3: 1078.
84. Kong W, He L, Coppola M, Guo J, Esposito NN, et al. MicroRNA-155 regulates cell survival, growth, and chemosensitivity by targeting FOXO3a in breast cancer. J Biol Chem 285: 17869-17879.
85. Lagos D, Pollara G, Henderson S, Gratrix F, Fabani M, et al. miR-132 regulates antiviral innate immunity through suppression of the p300 transcriptional co-activator. Nat Cell Biol 12: 513-519.
86. Xu D, Takeshita F, Hino Y, Fukunaga S, Kudo Y, et al. (2011) miR-22 represses cancer progression by inducing cellular senescence. J Cell Biol 193: 409-424.
87. Strum JC, Johnson JH, Ward J, Xie H, Feild J, et al. (2009) MicroRNA 132 regulates nutritional stress-induced chemokine production through repression of SirT1. Mol Endocrinol 23: 1876-1884.
88. Rivetti di Val Cervo P, Lena AM, Nicoloso M, Rossi S, Mancini M, et al. (2011) p63-microRNA feedback in keratinocyte senescence. Proc Natl Acad Sci U S A 109: 1133-1138.
89. Zhang J, Han C, Wu T (2012) MicroRNA-26a promotes cholangiocarcinoma growth by activating beta-catenin. Gastroenterology 143: 246-256 e248.
90. Chen K, Fan W, Wang X, Ke X, Wu G, et al. (2012) MicroRNA-101 mediates the suppressive effect of laminar shear stress on mTOR expression in vascular endothelial cells. Biochem Biophys Res Commun 427: 138-142.
91. Zhao G, Zhang JG, Liu Y, Qin Q, Wang B, et al. (2012) miR-148b functions as a tumor suppressor in pancreatic cancer by targeting AMPKalpha1. Mol Cancer Ther 12: 83-93.
92. Xu Q, Liu LZ, Qian X, Chen Q, Jiang Y, et al. (2012) MiR-145 directly targets p70S6K1 in cancer cells to inhibit tumor growth and angiogenesis. Nucleic Acids Res 40: 761-774.
93. Yamakuchi M, Yagi S, Ito T, Lowenstein CJ (2011) MicroRNA-22 regulates hypoxia signaling in colon cancer cells. PLoS One 6: e20291.
94. Dal Monte M, Landi D, Martini D, Bagnoli P (2013) Antiangiogenic role of miR-361 in human umbilical vein endothelial cells: functional interaction with the peptide somatostatin. Naunyn Schmiedebergs Arch Pharmacol 386: 15-27.
95. Yamakuchi M, Lotterman CD, Bao C, Hruban RH, Karim B, et al. (2009) P53-induced microRNA-107 inhibits HIF-1 and tumor angiogenesis. Proc Natl Acad Sci U S A 107: 6334-6339.
96. Zhu S, Pan W, Song X, Liu Y, Shao X, et al. (2011) The microRNA miR-23b suppresses IL-17-associated autoimmune inflammation by targeting TAB2, TAB3 and IKK-alpha. Nat Med 18: 1077-1086.
97. Ye H, Liu X, Lv M, Wu Y, Kuang S, et al. (2012) MicroRNA and transcription factor co-regulatory network analysis reveals miR-19 inhibits CYLD in T-cell acute lymphoblastic leukemia. Nucleic Acids Res 40: 5201-5214.
98. Iliopoulos D, Jaeger SA, Hirsch HA, Bulyk ML, Struhl K (2010) STAT3 activation of miR-21 and miR-181b-1 via PTEN and CYLD are part of the epigenetic switch linking inflammation to cancer. Mol Cell 39: 493-506.
99. Gantier MP, Stunden HJ, McCoy CE, Behlke MA, Wang D, et al. (2012) A miR-19 regulon that controls NF-kappaB signaling. Nucleic Acids Res 40: 8048-8058.
100. Kim SW, Ramasamy K, Bouamar H, Lin AP, Jiang D, et al. (2012) MicroRNAs miR-125a and miR-125b constitutively activate the NF-kappaB pathway by targeting the tumor necrosis factor alpha-induced protein 3 (TNFAIP3, A20). Proc Natl Acad Sci U S A 109: 7865-7870.
101. Lu Z, Li Y, Takwi A, Li B, Zhang J, et al. (2011) miR-301a as an NF-kappaB activator in pancreatic cancer cells. EMBO J 30: 57-67.
102. Steiner DF, Thomas MF, Hu JK, Yang Z, Babiarz JE, et al. (2011) MicroRNA-29 regulates T-box transcription factors and interferon-gamma production in helper T cells. Immunity 35: 169-181.
103. Wang M, Li C, Yu B, Su L, Li J, et al. (2012) Overexpressed miR-301a promotes cell proliferation and invasion by targeting RUNX3 in gastric cancer. J Gastroenterol.
104. Rodriguez A, Vigorito E, Clare S, Warren MV, Couttet P, et al. (2007) Requirement of bic/microRNA-155 for normal immune function. Science 316: 608-611.

105. Ding S, Liang Y, Zhao M, Liang G, Long H, et al. (2012) Decreased microRNA-142-3p/5p expression causes CD4+ T cell activation and B cell hyperstimulation in systemic lupus erythematosus. Arthritis Rheum 64: 2953-2963.
106. Nazarov PV, Reinsbach SE, Muller A, Nicot N, Philippidou D, et al. (2013) Interplay of microRNAs, transcription factors and target genes: linking dynamic expression changes to function. Nucleic Acids Res 41: 2817-2831.
107. Ma F, Xu S, Liu X, Zhang Q, Xu X, et al. (2011) The microRNA miR-29 controls innate and adaptive immune responses to intracellular bacterial infection by targeting interferon-gamma. Nat Immunol 12: 861-869.
108. He M, Wang QY, Yin QQ, Tang J, Lu Y, et al. (2013) HIF-1alpha downregulates miR-17/20a directly targeting p21 and STAT3: a role in myeloid leukemic cell differentiation. Cell Death Differ 20: 408-418.
109. Chen L, Yang Q, Kong WQ, Liu T, Liu M, et al. (2012) MicroRNA-181b targets cAMP responsive element binding protein 1 in gastric adenocarcinomas. IUBMB Life 64: 628-635.
110. Liao Y, Lonnerdal B (2010) Global microRNA characterization reveals that miR-103 is involved in IGF-1 stimulated mouse intestinal cell proliferation. PLoS One 5: e12976.
111. Zhou J, Wang KC, Wu W, Subramaniam S, Shyy JY, et al. (2011) MicroRNA-21 targets peroxisome proliferators-activated receptor-alpha in an autoregulatory loop to modulate flow-induced endothelial inflammation. Proc Natl Acad Sci U S A 108: 10355-10360.
112. Lee EK, Lee MJ, Abdelmohsen K, Kim W, Kim MM, et al. (2010) miR-130 suppresses adipogenesis by inhibiting peroxisome proliferator-activated receptor gamma expression. Mol Cell Biol 31: 626-638.
113. Lal A, Navarro F, Maher CA, Maliszewski LE, Yan N, et al. (2009) miR-24 Inhibits cell proliferation by targeting E2F2, MYC, and other cell-cycle genes via binding to "seedless" 3'UTR microRNA recognition elements. Mol Cell 35: 610-625.
114. Sachdeva M, Zhu S, Wu F, Wu H, Walia V, et al. (2009) p53 represses c-Myc through induction of the tumor suppressor miR-145. Proc Natl Acad Sci U S A 106: 3207-3212.
115. Buechner J, Tomte E, Haug BH, Henriksen JR, Lokke C, et al. (2011) Tumour-suppressor microRNAs let-7 and mir-101 target the proto-oncogene MYCN and inhibit cell proliferation in MYCN-amplified neuroblastoma. Br J Cancer 105: 296-303.
116. Fan Q, He M, Deng X, Wu WK, Zhao L, et al. (2012) Derepression of c-Fos caused by MicroRNA-139 down-regulation contributes to the metastasis of human hepatocellular carcinoma. Cell Biochem Funct.
117. Loftus JC, Ross JT, Paquette KM, Paulino VM, Nasser S, et al. (2012) miRNA expression profiling in migrating glioblastoma cells: regulation of cell migration and invasion by miR-23b via targeting of Pyk2. PLoS One 7: e39818.
118. Liu M, Tang Q, Qiu M, Lang N, Li M, et al. (2011) miR-21 targets the tumor suppressor RhoB and regulates proliferation, invasion and apoptosis in colorectal cancer cells. FEBS Lett 585: 2998-3005.
119. Ma L, Teruya-Feldstein J, Weinberg RA (2007) Tumour invasion and metastasis initiated by microRNA-10b in breast cancer. Nature 449: 682-688.
120. Zhang J, Ying ZZ, Tang ZL, Long LQ, Li K (2012) MicroRNA-148a promotes myogenic differentiation by targeting the ROCK1 gene. J Biol Chem 287: 21093-21101.
